# Robust Double Emulsions for Multicolor Fluorescence-Activated Cell Sorting

**DOI:** 10.1021/acs.analchem.4c02363

**Published:** 2024-09-04

**Authors:** Yun Ding, Giada Zoppi, Gaia Antonini, Roger Geiger, Andrew J. deMello

**Affiliations:** † Institute for Chemical and Bioengineering, Department of Chemistry and Applied Biosciences, ETH Zürich, 8093 Zürich, Switzerland; ‡ Institute for Research in Biomedicine, Faculty of Biomedical Sciences, Università della Svizzera italiana, 6500 Bellinzona, Switzerland; § Institute of Oncology Research, Faculty of Biomedical Sciences, Università della Svizzera italiana, 6500 Bellinzona, Switzerland

## Abstract

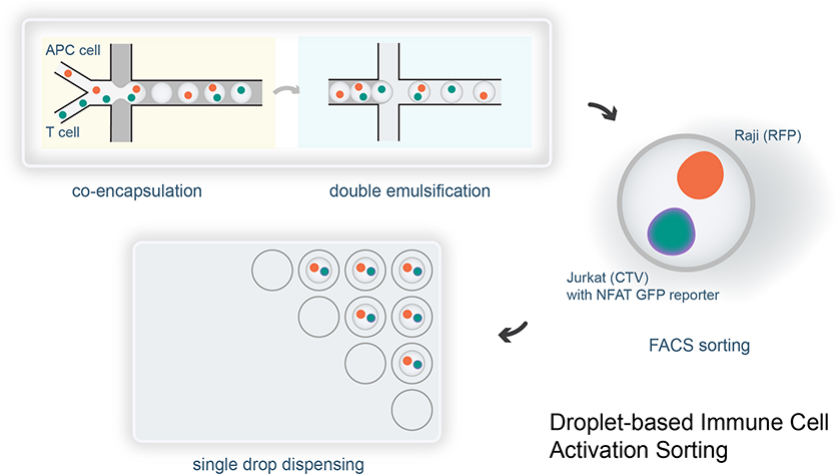

Cell–cell interactions are essential for the proper functioning of multicellular organisms. For example, T cells interact with antigen-presenting cells (APCs) through specific T-cell receptor (TCR)–antigen interactions during an immune response. Fluorescence-activated droplet sorting (FADS) is a high-throughput technique for efficiently screening cellular interaction events. Unfortunately, current droplet sorting instruments have significant limitations, most notably related to analytical throughput and complex operation. In contrast, commercial fluorescence-activated cell sorters offer superior speed, sensitivity, and multiplexing capabilities, although their use as droplet sorters is poorly defined and underutilized. Herein, we present a universally applicable and simple-to-implement workflow for generating double emulsions and performing multicolor cell sorting using a commercial FACS instrument. This workflow achieves a double emulsion detection rate exceeding 90%, enabling multicellular encapsulation and high-throughput immune cell activation sorting for the first time. We anticipate that the presented droplet sorting strategy will benefit cell biology laboratories by providing access to an advanced microfluidic toolbox with minimal effort and cost investment.

## Introduction

Droplet-based microfluidics has dramatically reshaped the execution of high-throughput and massively parallel biological experiments over the past decade.^1−3^ Innovations such as droplet digital PCR (ddPCR),^4^ droplet-based single-cell RNA sequencing (DB scRNA-seq),^5,6^ and fluorescence-activated droplet sorting (FADS)^7^ have made impacted genetic analysis, transcriptome sequencing, and biological screening. These technologies utilize the unique properties of microfluidically produced droplets to partition large numbers of small assay/reaction volumes, enabling the rapid generation and analysis of substantial data sets. For example, ddPCR has revolutionized the quantitative analysis of DNA targets, providing unmatched sensitivity for genetic and infectious disease detection.^8−11^ Additionally, it has become an essential tool in monitoring circulating tumor cells in cancer recurrence management.^12−14^ Similarly, DB scRNA-seq has dramatically reduced transcriptome sequencing costs per cell, allowing the interrogation of tens of thousands of individual cells in a single run.^15^ Such approaches are now integral in mapping gene expression during biological development and identifying targets for precision medicine.^16−19^ FADS, capable of detecting “one in a million” events,^20,21^ offers a compelling alternative to traditional microplate-based screening methods, reducing reagent use and enhancing analytical throughput.^22,23^ Interestingly, compared to FADS, ddPCR and DB scRNA-seq have seen far broader commercialization (with the Bio-Rad QX ONE ddPCR system and 10x Genomics Chromium X single cell platform being leading examples) and application, with FADS being largely confined to specialized laboratories. This discrepancy stems from the complex nature of dedicated droplet sorter systems, which integrate multiple advanced components such as precise fluid control, high-speed droplet imaging, and sophisticated sorting and dispensing mechanisms, limiting their accessibility and use.^24,25^

Although the idea of repurposing commercial fluorescence-activated cell sorting (FACS) instruments for sorting double emulsions (DEs) was suggested twenty years ago,^26,27^ it has only recently emerged as a convenient alternative to specialized droplet sorters.^28−31^ This cost-effective approach simplifies the creation and sorting of DEs, and is gradually becoming standard practice.^32−34^ The current trend in FACS DE generation has moved away from complex 3D fluidic channels^28^ or coaxial capillaries,^35^ instead adopting relatively simple single layer microfluidic structures.^36,37^ Such devices employ localized coatings^38^ to create distinct hydrophobic and hydrophilic zones, enabling the continuous production of water-in-oil (W/O) droplets and subsequent W/O/W DEs within a single processing step. Such DEs are compatible with FACS sorters and allow for selection based on the fluorescence properties of the encapsulated cells.

Recently reported DE workflows for FACS sorting can be highly effective, with detection rates ranging from 85% to 95% when applied to single-molecule nucleic acid screening.^32^ This surpasses the sub-50% rates typically reported in earlier studies.^28,39^ However, efficiencies decrease when applied to single-cell DEs, with detection rates dropping to 63.8%.^33^ This drop in performance highlights the method’s limitations in managing larger payloads. Moreover, the prevalent one-step DE formation approach has considerable practical drawbacks. First, the method lacks adaptability and is incompatible with standard droplet manipulations such as merging, splitting, and pico-injection.^3^ Second, the use of excessive oil results in a thick oil shell around the DE, restricting internal droplet size and making the method unsuitable for encapsulating multiple cells. Indeed, as noted by Zinchenko and co-workers, to ensure stable droplet break-off during FACS sorting, the particle size should not exceed one-third of the nozzle diameter.^39^ This means that when using a typical a 70 μm FACS nozzle, the maximum DE diameter will be 23 μm. Third, in the one-step method, the operational parameter restrictions often lead to the production of empty DEs (pure oil droplets) or fail to achieve the precise total DE volume necessary for optimal FACS sorting.^40^ Finally, the use of localized coatings on chips can complicate manufacturing and reduce accessibility for general users.

In the current work, we aim to address the aforementioned limitations by defining a universally applicable, adaptable, and easy-to-operate workflow for applications involving multiple mammalian cells. Interestingly, at the time of writing, no study has demonstrated the successful encapsulation of two mammalian cells within double emulsions, and subsequent FACS-based DE sorting based on a fluorescence phenotype. In this regard, there exists a pressing need for methods able to efficiently screen complex cellular interactions, such as T cell activation in response to antigen-presenting cell (APC) stimulation.^41−43^ Accordingly, we introduce an accessible and flexible DE microfluidic workflow that delivers high-quality DEs. Importantly, the workflow can be easily adopted by cell biologists and achieves DE detection rates exceeding 90% in multicell applications. To demonstrate the utility of the workflow, we show immune cell activation sorting using DE FACS with triple positive color gating for the first time. Although initially optimized for coencapsulation and coculture of two mammalian cell types, our methodology is applicable to single molecules, bacteria, and larger cells, due to its reliability and the provision of a large internal droplet size. By presenting this workflow, we hope to make advanced droplet sorting accessible to a broader spectrum of biological research laboratories.

## Materials and Methods

### Cell Preparation

Cells, including peripheral blood mononuclear cells (PBMCs) sourced from donated blood compliant with Swiss federal regulations, were cultured at 37 °C with 5% CO_2_. CD4^+^ and CD8^+^ T cells, enriched using magnetic microbeads (Miltenyi Biotec, Adliswil, Switzerland), along with Raji, Jurkat D1.1, HEK293T, and K562 cell lines (ATCC, Manassas, USA), were maintained in appropriate media. Primary human B cells, isolated similarly, were immortalized with Epstein–Barr Virus and cultured for expansion. For lentiviral production, HEK293T cells were transfected with pCDH vectors (System Biosciences, Palo Alto, USA) and the necessary packaging plasmids (psPAX2 and pMD2.G from Addgene) to produce viral particles, which were then harvested and concentrated using the Lenti-X concentrator (TakaraBio, Kusatsu, Japan). These viral particles were used to transduce T cells and Raji cells, followed by culture and analysis using flow cytometry. Cell labeling utilized specific dyes (CellTrace Violet, Cell Trace Far Red, and CellTrace CFSE from Thermo Fisher Scientific, San Diego, USA), and an NFAT-eGFP reporter system was developed through cloning and plasmid modification and verified by Sanger sequencing (Microsynth AG, Balgach, Switzerland). Detailed protocols and additional methodological specifics are provided in the Supporting Information.

### Microfluidic Device Fabrication and Operation

Microfluidic devices for droplet generation and double emulsion conversion were fabricated in polydimethylsiloxane (PDMS) via standard soft lithography. Microchannel surfaces were treated to be either hydrophobic (for droplet generation) or hydrophilic (for DE conversion). Detailed fabrication protocols are provided in the Supporting Information.

Cell suspensions were filtered and then coflowed with oil through the droplet generation device at optimized flowrate ratios to form water-in-oil droplets of the desired size. After storage, these droplets were introduced into the DE conversion device, where they were re-encapsulated in an aqueous stream containing surfactant to form DEs. The flowrate ratios of the droplets and aqueous buffer were optimized, along with surfactant compositions, to enable stable thin-shell DE formation. Specific steps, materials, flowrates, and surfactant details are detailed in the Supporting Information.

### DE Sorting and Analysis

DEs containing coencapsulated cells were diluted in PBS, resuspended in FACS tubes, and sorted either collectively into another FACS tube or individually into a 96-well plate using incrementally adjusted droplet delays to optimize sorting efficiency. DEs in well plates were imaged using an ImageXpress Micro 4 high-content microscope (Molecular Devices, San Jose, USA). FACS data were analyzed using FlowJo v10 software (BD Biosciences, Ashland, USA). Co-encapsulation efficiency was evaluated by imaging DEs containing primary T cells (GFP) and Raji cells (CellTrace Violet) at different cell concentrations. ImageJ/Fiji (https://fiji.sc) was utilized for droplet counting and analysis. The effect of external osmolarity on coencapsulated cells was investigated by incubating DEs across various PBS concentrations, with pre- and postincubation imaging to examine DE integrity and FACS data to evaluate sorting impact. Detailed imaging setups, descriptions of the cell lines used, and other experimental conditions are provided in the Supporting Information.

## Results and Discussion

### Microfluidic Workflow

**Figure 1:**
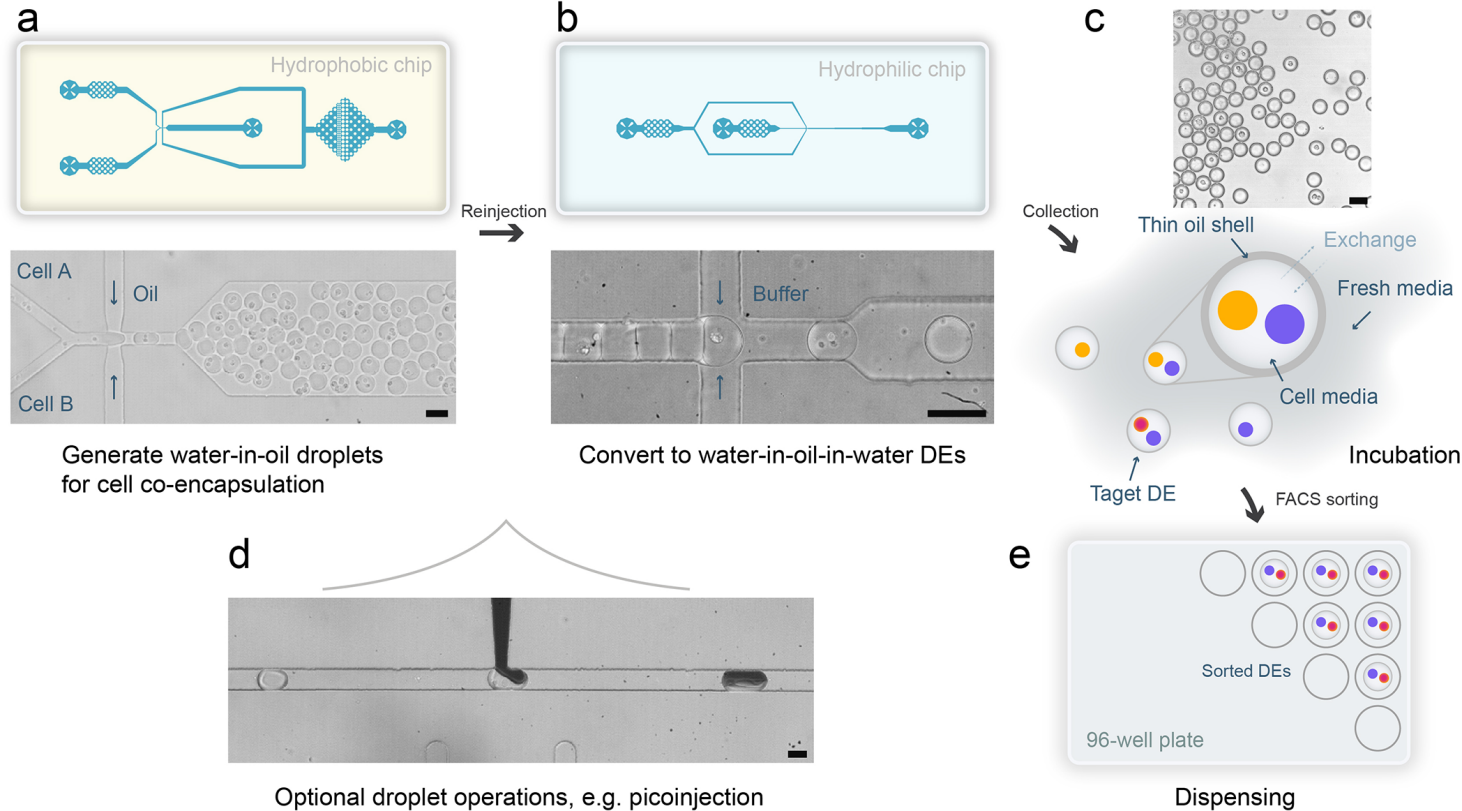
Co-cell culture DE sorting workflow. The workflow begins with a hydrophobic PDMS microfluidic device that is used to coencapsulate cells in water-in-oil droplets (a). The collected droplets are then reinjected into a hydrophilic PDMS device to form DE drops (b). Both schematics and brightfield images of the devices are shown. Videos demonstrating each step are available as Video S1 (coencapsulation) and Video S2 (DE formation). The resulting thin-shell DEs are collected for incubation (c), allowing the DE buffer to be replaced with fresh cell culture media (surfactant-free) to support cell culture without damaging the DE drops. After incubation, DE drops are sorted using a commercial flow cytometer in “single cell” sorting mode, which automatically dispenses individual target cell combinations into the wells of a microplate (e). The average thickness of the DE shells in (c) was measured to be 3.6 μm. The 2-step DE generation process also allows for additional droplet operations, such as pico-injection (d); here 10% blue ink is used for visualization purposes. Actual reagents include lysis buffer and RT-mix, depending on specific cell screening needs.^74,75^ The scale bars in all images are 50 μm.

Understanding the challenges faced by cell biology laboratories, especially those with limited experience in microfluidic technologies, guided our development of an accessible double emulsion workflow (refer to project background in Supporting Information). Our primary objective was to create a user-friendly system that simplifies complex processes and enhances experimental flexibility. This is achieved by separating the generation of W1/O droplets and W1/O/W2 DEs across two single-layer PDMS devices. An overview of the complete workflow is presented in Figure 1. Our modular design^39^ offers three main advantages. First, it eliminates the complexities and synchronization required in one-step DE generation methods,^40,44^ allowing for independent control over internal droplet size.^45^ This resolves issues where adjusting droplet size compromises optimal FACS sorting. The user can fine-tune parameters to ensure precise droplet contents and then adapt the system to experimental need. Second, chip fabrication is straightforward, requiring no selective local surface treatments. This means that laboratories can easily access devices that produce DEs tailored to their specific requirements. Third, the approach retains configurational flexibility, which is especially beneficial when performing complex biological experiments, supporting unit operations such as merging, injection (Figure 1d), and thermal cycling on droplets prior to conversion into DEs.^4,46^ The complete workflow consists of coencapsulation of cells, double emulsification, droplet incubation, and FACS sorting. Initially, fluorinated oil droplets of optimal size for single and multicellular encapsulation are generated. These oil droplets are then converted into DE drops suitable for cell culture and subsequent FACS processing. Finally, the DEs are processed and sorted using FACS.

### Co-Encapsulation of Two Cells

Droplet-assisted cellular sorting widens the spectrum of quantifiable cellular events. Instead of relying solely on signals from a cell’s interior or its surface, the droplet volume may be used to capture cell-secreted signals for more comprehensive screening assays.^24,47^ Further, the ability to accommodate multiple cells within a single droplet provides a powerful tool for studying cell-cell interactions.^48^ Indeed, such a strategy holds great potential for exploring immune therapies through the high-throughput screening of cellular immune responses.^49^ As such, our DE sorting workflow was particularly designed for a coculture system.

To enable the coencapsulation of two cell populations, we used a PDMS microfluidic device (Figure 1**a**). The design incorporates a flow-focusing geometry to generate monodisperse droplets having diameters between 40 and 50 μm. This size range not only accommodates cellular nutrients and promotes cell-cell interaction but also ensures compatibility with the 130 μm nozzle commonly used in FACS instruments. Crucially, a 50 μm diameter represents the upper limit for successful sorting with a 130 μm FACS nozzle.^32^ Further details of the design parameters can be found in the Supporting Information. In a typical experiment, two cell streams are injected at a flowrate of 3 μL/min each, converging just before an orifice where they are focused by an oil flow of 10 μL/min to form droplets. These droplets are subsequently collected in a 1 mL syringe and prepared for subsequent reinjection into the DE conversion device.

**Figure 2:**
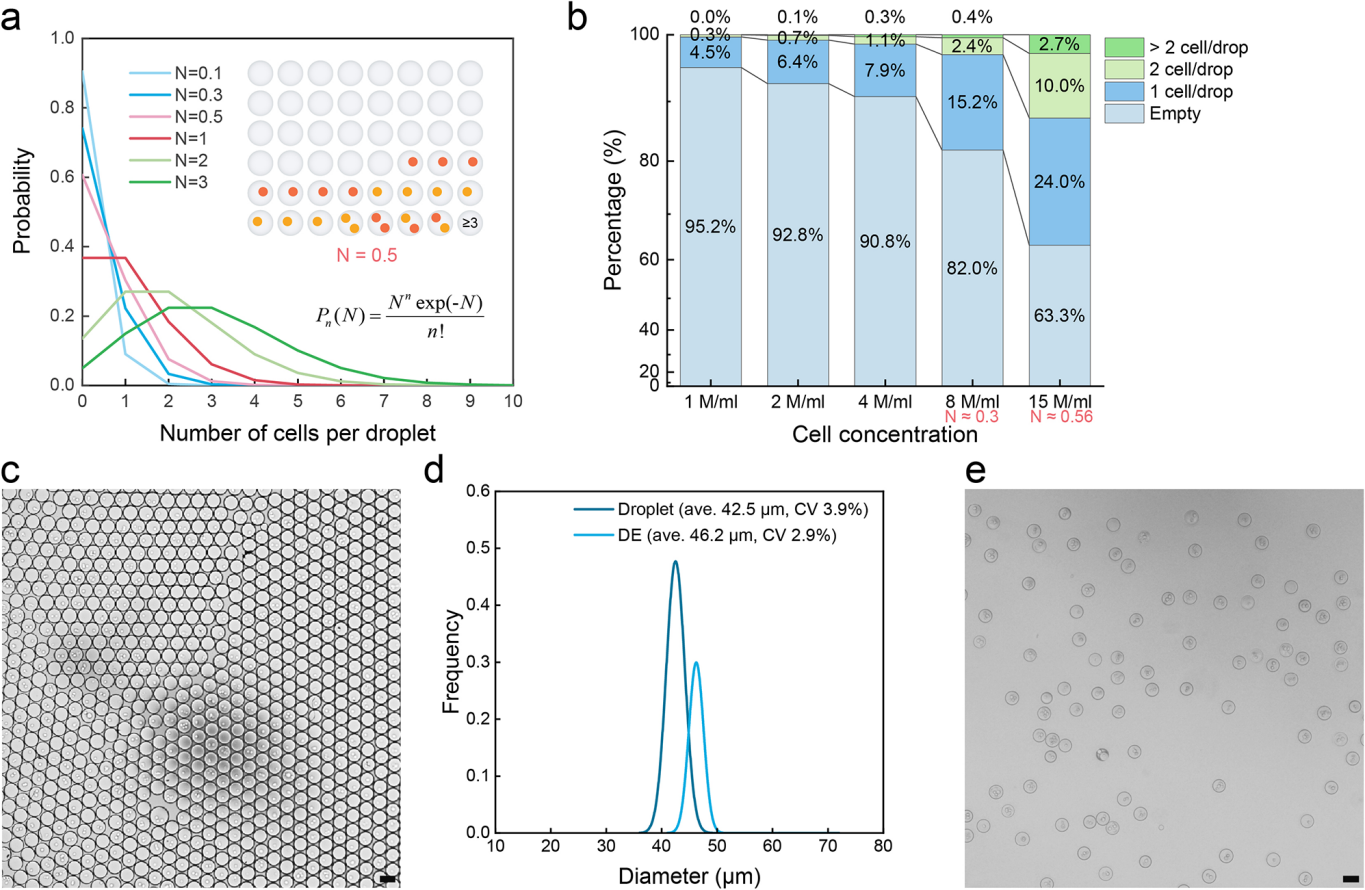
Single-cell coencapsulation in droplets. (a) Poisson statistical predictions for single cell coencapsulation at varying cell densities show that with an average *N* of 0.5, over two in every 48 droplets will include both cell types. *N* represents the average number of total cells in droplets, and *P* signifies the probability of encountering *n* cells in a droplet. (b) The experimental occupancy statistics for coencapsulating cells (GFP-expressing T cells and CTV-stained Raji cells) in droplets. Input cell concentrations of 8 or 15 million/ml (for both cell types) are routinely employed in the current experiments, giving overall *N* values of 0.3 and 0.5, respectively. These *N* values, referenced by ddPCR, represent a suitable working range for single cell encapsulations, balancing coencapsulation efficiency and statistical accuracy.^76^ (c) Representative microscopic image of coencapsulated droplets. (d) Droplet and subsequent DE drop size distributions, highlighting a high degree of monodispersity. The average size of a daughter DE drop increases by 3.7 μm compared to its mother droplet. (e) Post-FACS sorted DE drops imaged under a microscope showcase unaltered structural integrity. The scale bars in all images are 50 μm.

Encapsulation efficiencies were assessed based on Poisson statistics (Figure 2a,b). Poisson statistics are a useful tool to access droplet occupancy, with optimal results being obtained when the average number of cells per droplet is maintained between 0.3 and 0.6. This ensures that 3.8% to 4.9% of droplets will contain one cell of type A and one cell of type B. Such a selection balances coencapsulation efficiency (i.e., ensuring the optimal possible single cell to single cell correspondence) and accuracy (minimizing instances of one-to-many or many-to-many cell combinations). A representative image of coencapsulated droplets is shown in Figure 2c.

### Conversion of Droplets to Thin-Shell DEs

Thin-shell DEs have potential benefits. First, they facilitate material exchange across the oil layer, which could benefit cell culture (Figure S2a).^50^ Second, they enhance hydrodynamic resilience, which could facilitate the fluid sorting process. As shown in previous studies,^51−54^ a thin oil shell renders W1/O/W2 DEs more resilient during fluid transport and processing. Specifically, under a given shear rate, DEs with a thinner oil shell experience smaller deformation displacement. This is attributable to the higher internal shear stress generated by the thin shell, which resists relative motion between adjacent layers (see Figure S2b, top, illustrating a DE in shear flow).^55,56^ Similarly, when subjected to a rotating flow field, a thin oil shell reduces the deflection angle between the oil phase and internal water phase, mitigating ’shock’ from variations in Laplace pressure (see Figure S2b, bottom, demonstrating a DE within a rotating flow).^56,57^

The process of creating thin-shell DEs involves the minimization of excess oil introduction during cell coencapsulation. By utilizing a ’close packing’ reinjection procedure,^58^ we minimize oil content prior to DE formation. The reinjection device includes a droplet and buffer inlet and a DE outlet (Figure 1b). We employ a single layer PDMS device, similar to the one used for coencapsulation, but with an added hydrophilic surface. The reinjection channel is designed to have a cross-sectional area that is slightly smaller than the droplet size, facilitating a pearl necklace-like arrangement of droplets. This enables efficient conversion of individual droplets into thin-shell DEs, while preventing double or multiple droplet encapsulation in a single DE. Our DEs feature remarkably thin oil shells, averaging 3.6 μm in thickness (Figures 1b,c and 2d). This not only enhances stability but also ensures that the overall size of each DE remains under 50 μm and is compatible with the size constraints set by a 130 μm FACS nozzle.^32^ Finally, and in addition to the basic DE device shown in Figure 1b, an extended chip that can accommodate a wide range (35–75 μm) of input droplet sizes was also fabricated (Figure S1c).

It is important to note that although this is not the first example of a decoupled method of FACS-compatible double emulsion production, it is the first to highlight that DEs can be stably formed without the introduction of space oil. This aspect has significant practical value for the end-user, as it allows the production of high quality DEs for FACS. For instance, both Zinchenko and co-workers^39^ and the commercial Dolomite Bio system^59^ have previously used a two-step method but introduce a spacer oil in the second step to prevent the formation of doublets. In the former study DEs were so large that they had to be osmotically shrunk to comply with the size limitations imposed by FACS nozzles. In the latter, the Dolomite Bio system produces DEs with an outer diameter of 30 μm and an inner aqueous core typically ranging between 15 and 17 μm. While these dimensions are suitable for encapsulating smaller volumes, our system permits a significantly larger internal space (a nearly 16 times increase), facilitating the encapsulation and culturing of larger cells or cell clusters, which are crucial for targeted applications in cellular interaction studies.

### Stability Testing

Co-cell culture in DEs is challenging since the generation of detectable signals from a stimulated cell can take several hours or even days.^60,61^ Remarkably, our DEs display exceptional stability postcreation. Under “standard” conditions (where the cell culture medium is encapsulated within the DEs, and these are in turn surrounded by a PBS buffer, all maintained at room temperature or refrigerated at 4 °C) DEs maintain their integrity for at least a year without rupture or fusion. They can withstand physical manipulations such as vortexing and pipetting. The corresponding assessment is provided in Figure S3. Additionally, we evaluated the stability of DE cultures under conditions of varying ionic strength (Figure S4). Results confirmed the robustness of our DEs, making them suitable for cell encapsulation and cell culture.

Recognizing the stability of DEs under FACS sorting conditions, we subsequently conducted experiments involving the application of electric fields and the use of osmotic variations. We first passed the DEs through a microfluidic channel while applying either an alternating electric field (up to 10 kHz and 1 kV) or a constant electric field of 1 kV using an apparatus with a configuration analogous to that depicted in Figure S5a. No deformation or breakage of DEs was observed for all tested conditions (data not shown). Subsequently, we exposed DEs in bulk to an antistatic gun (Milty Zerostat 3), a tool known for its efficiency in generating a focused electric field able to rupture droplets.^62^ Interestingly, no DE damage was observed. We attribute the exceptional electric field stability to the shielding effect offered by the external (conductive) buffer.^63^ To test this theory, DEs containing two closely contacted droplets were passed through an electric field in a constricted area (Figure S5a and Video S3). Despite the susceptibility of water-in-oil droplets to fusion in electric fields due to transient interface instabilities,^64−66^ no fusion was observed in our experiments. In comparison, normal droplets merged in a microfluidic channel under the same electric condition (Figure S5b), indicating the absence of an internal electric field in DEs. To conclude, our DEs exhibit remarkable stability under electric fields, making them well-suited for FACS processing. The integrity of FACS sorted DEs can be found in Figure 2e.

### Sorting of Co-Cell DEs

We next sought to determine if DEs could be successfully sorted using a commercial FACS. To test this, we encapsulated CTV-labeled Raji cells and GFP-expressing T cells into droplets followed by double emulsification. The resulting DEs were resuspended in PBS and sorted using a FACS Aria III instrument equipped with a 130 μm nozzle, implementing automated sample shaking to mitigate DE aggregation. As observed in Figure S6a, DEs formed a subpopulation that was distinct from fragments (debris or nontarget particles). After selection of the DE population, we detected a strong signal in the 405 nm fluorescence channel and a modest signal at 488 nm (Figure S6b), confirming the presence of CTV-labeled cells, GFP-expressing cells, or both within the DEs. In contrast, no fluorescence was detected when gating the fragment populations (Figure S6c). Notably, over 90% of detected events were assigned to DEs, significantly higher than the 63.8% rate^33^ observed in the previous study involving single-cell encapsulations. This highlights the high quality of the DEs for cocell encapsulations and underscores our method’s effectiveness in enhancing sorting performance. In addition, it should be noted that the scattering parameters (i.e., FCS and SSA) generally only provide information on the size and shape of the detected species, and do not reveal internal characteristics. For example, it is impossible to confirm whether a DE houses cells or is empty by only examining scatter. Interestingly, we can differentiate the number of droplets within a DE using scatter signals, as shown in Figure S7. This analysis distinguishes “Chaos” DEs into three subpopulations based on core counts, indicating single-core, double-core, and multicore DEs.

### DE Breakage

DEs provide a confined environment for isolating and manipulating small samples. However, if the encapsulated samples need further processing, the ability to robustly break the DE is requisite. DEs can be effectively ruptured by temporarily placing them on a dry surface or using a demulsifier such as perfluorooctanol,^32,67^ as illustrated in Figure S8a. Additionally, we developed a new simple method to rupture DEs using freezing and thawing. Here DEs are placed in a −80 °C freezer for 15 min, followed by rapid thawing in a 37 °C water bath. Using this method, we found that all DEs ruptured (Figure S8b). Osmotic swelling is another potential method to release DEs. This method depends heavily on the difference between internal and external osmotic pressure, DE size, and the thickness of the oil shell.^54,68^ We found that placing DEs in DI water did not cause rupture, although the DE diameter increased by 20% (Figure S4d). However, when DEs contained 16% OptiPrep (a common density medium to balance cells), approximately 67% of the DEs ruptured, with their diameters increasing to over three times the original size (Figure S8c).

### Optimization Strategies for DE Sorting

**Figure 3:**
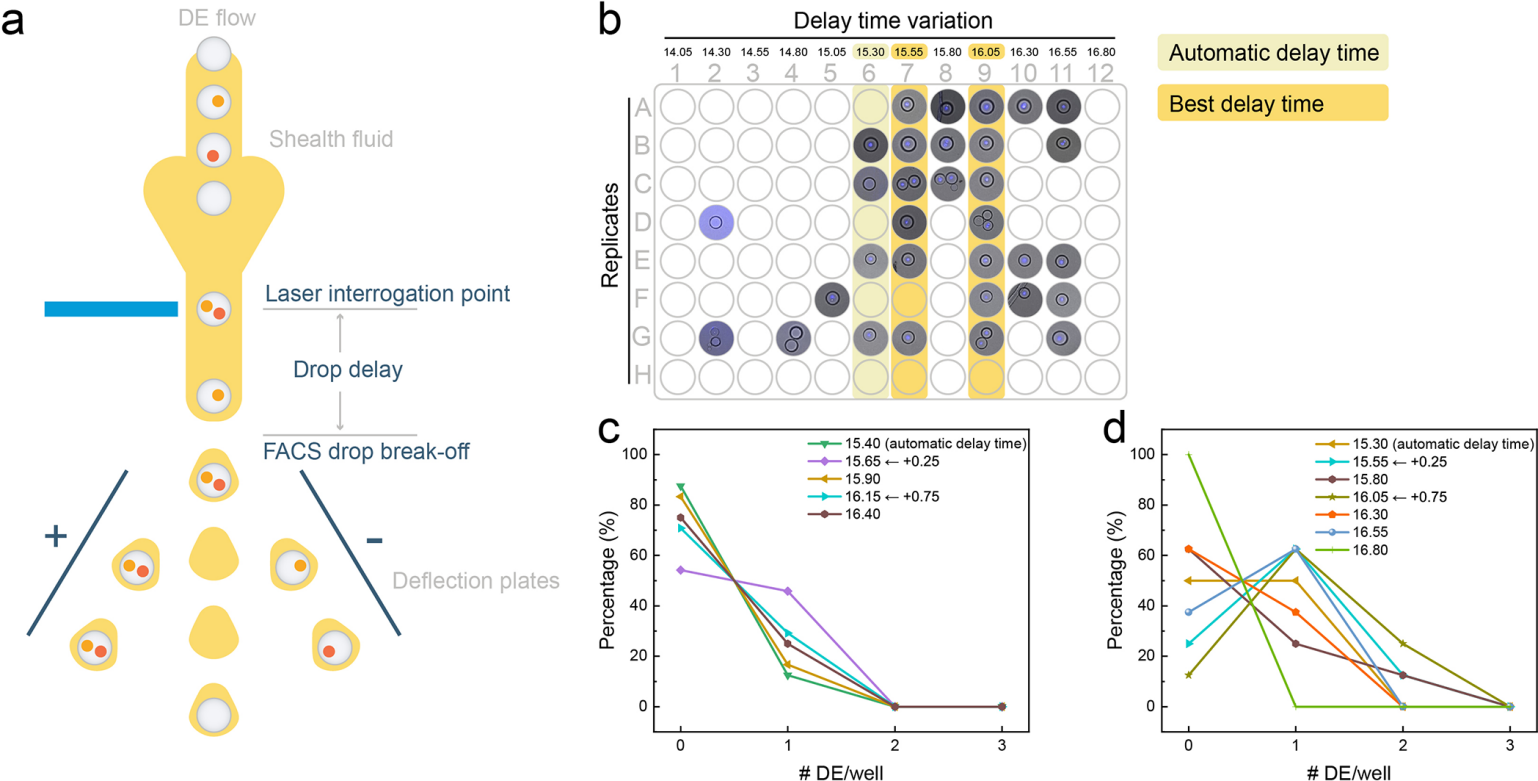
Control of drop delay settings on the FACS instrument to accurately target deflection and sorting. (a) Schematic describing the impact of drop delay. (b) Visual comparison of drop delay effects on a 96-well plate, with each well programmed to receive two sorted DEs. Delay times range from 14.05 to 16.80 in 0.25 intervals. The two most effective delay times (15.55 and 16.05, corresponding to +0.25 and +0.75 based on the automatic delay time) are highlighted. (c) Statistical analysis of single DE sorting at different delay times, with each well set to receive one DE. (d) Statistical analysis of “two for one” sorting at different delay times, aiming for one DE per well. It should be noted that the droplet delay time is measured in droplet cycles rather than seconds. For example, a drop delay of 15 indicates that the instrument must wait for 15 FACS drops after detecting a gated event before applying an electronic charge.

When sorting individual DEs into 96-well microplates using FACS, management of drop delay is crucial to ensure the accurate collection of gated events. Drop delay describes the time required for species to move from the laser interrogation point to the break-off point, where the stream is transformed into charged droplets for deflection (Figure 3a). An incorrect drop delay will lead to inaccurate sorting outcomes, either through the rejection of “targeted” species (empty sorting) or the collection of unwanted species (mistaken sorting). While FACS instruments integrate automated drop delay control, which can be adjusted via calibration by microbeads (in our case, 6 μm Accudrop Beads), this is suboptimal for DE sorting. Such a limitation arises because DEs are larger and slower than cells. Indeed, when using automatic delay control, we achieved only a 10% success rate when loading single DEs into wells. To optimize delay compensation, we systematically adjusted the time in increments of 0.25 droplet cycles. Our analysis revealed that the +0.25 and +0.75 settings provided the best performance, with the +0.25-setting yielding the highest loading rate (50%) for single DEs (Figure 3c).

Optimizing the drop delay is essential for accurate FACS sorting, yet inherent performance limitations will restrict the efficiency of single DE dispensing across different instrument models. To overcome this issue, we adopted a ’two-cell’ sorting mode, with the FACS instrument dispensing two DEs into each well. This strategy improved the likelihood of wells containing exactly one DE to over 60%, with approximately 10% of wells containing two DEs, thereby enhancing overall sorting success (Figure 3d). As part of our “two-for-one” mode evaluation, we systematically incremented the drop delay by 0.25 units across a 96-well plate, with each column assigned a specific delay (Figure 3b). The most effective delay times for this mode were found to be 15.55 and 16.05 units, corresponding to +0.25 and +0.75 increments from the baseline automatic delay. The recurrence of these increments as optimal in both “two-for-one” and “single” DE modes suggests an underlying consistency in the instrument’s handling of DEs, indicating that these delay adjustments compensate for the dynamics unique to DEs, and can serve as a general guide for precision in DE sorting.

### Comparative Analysis of Cell Labeling Techniques in DE Screening

Direct cell labeling, favored for its high signal-to-noise ratio, streamlined labeling workflows and compatibility across a range of cell types, often takes precedence over methods such as fluorescence protein expression.^69^ Indeed, fluctuations in emission yield can significantly influence the efficiency of DE screening, as shown in Figure S6. Here, CTV-labeled Raji cells and GFP-expressing T cells were initiated at equivalent concentrations of 8 million/ml, each at a final concentration of 4 million/ml in the costream and at an *N* value of 0.3. According to Poisson statistics, we expected approximately 13.93% of DEs to exhibit fluorescence from either cell type. Our FACS results aligned closely with this expectation for CTV-labeled Raji cells 13.73% (Q1 + Q2), but GFP-expressing T cell DEs fell drastically short at only 0.7% (Q3+Q2). Here, direct labeling produces superior signals compared to intrinsic fluorescent protein expression. Additionally, the DE shell appears to hinder fluorescence detection. Even though all T cells, prior to encapsulation, undergo FACS sorting and culture expansion, theoretically making them all GFP signal carriers, after encapsulation into DEs only 5% (0.7%/13.93%) of T cells could be detected. In addition to cell labeling techniques, we also investigated the regulation of the internal core size through osmotic pressure control as a potential approach to enhance biological signal detection. Our findings suggest that the benefits of this approach are not straightforward and depend on specific experimental conditions and the nature of the biomarkers involved. Further details of this analysis are provided in Figure S9.

To further investigate the influence of direct labeling methods on screening efficiency, we compared surface labeling with whole-cell staining methods. Specifically, we coencapsulated equal concentrations of APC-labeled (anti-CD45-APC) T cells and CTV-stained Raji cells. The APC fluorophore binds to the cell membrane by targeting the CD45 surface antigen, whereas the CTV dye binds across the whole cell wherever free amines are present. The FACS screening results, presented in Figure S10, revealed the similarity in fluorescence detection efficiency and gating ability between the two methods, with detection rates exceeding 5% in both instances. These findings suggest that the choice between surface labeling and whole-cell staining may not substantially affect screening efficiency. Of course, the optimal labeling strategy should be determined by other factors, such as cell type, experimental conditions and specific downstream analytical requirements.

### Sorting DEs with Three Fluorescent Channels and Its Application in Immune Cell Activation

**Figure 4:**
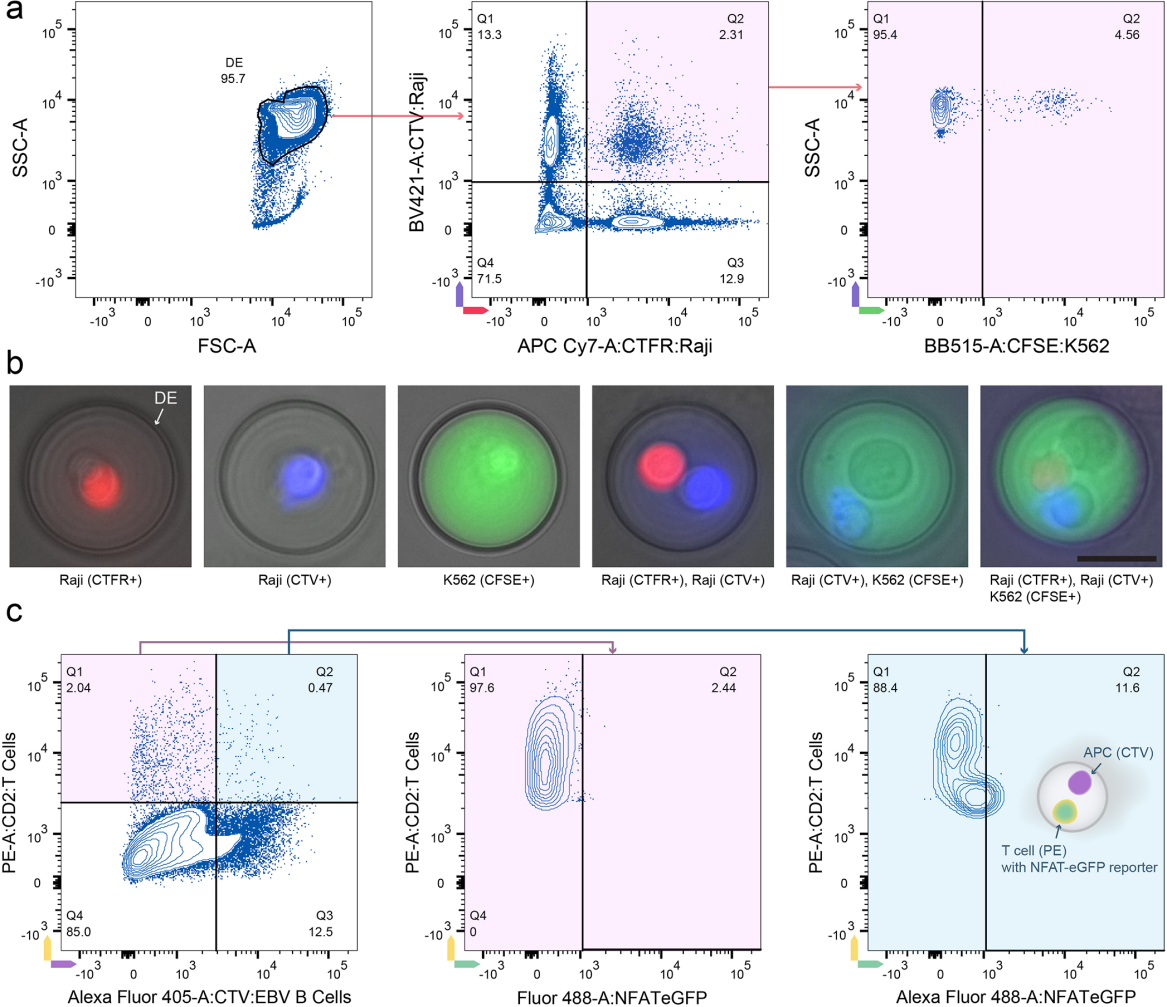
Multicolor detection and immune cell activation sorting using DEs. (a) Flow cytometry analysis of DEs containing different cell types. Raji cells stained with CTFR and CTV, respectively, are premixed at a ratio of 1:1 (8 million/ml) and then coencapsulated with CFSE-stained K562 cells (8 million/mL). The FSC-SSC scatter plot (left) shows a majority DE population (95.7%). Scatter plots gated on DE events display CTFR (Raji cells) vs CTV (Raji cells) (middle), and further gating identifies DEs containing CFSE (K562 cells) (right), highlighting the multicolor detection capability. (b) Representative microscopy images (overlapping brightfield and fluorescence) of sorted DEs show individual and combined fluorescent signals from Raji cells (CTFR+ and CTV+), K562 cells (CFSE+), and their combinations, demonstrating successful coencapsulation and detection of multiple cell types. Note that the CFSE dye is exceptionally bright, creating the perception of dye diffusion throughout the DE under fluorescence examination. The scale bar is 25 μm. (c) Immune cell activation assay. EBV-specific CD4^+^ T cells, transduced with an activation reporter and labeled with anti-CD2-PE antibody, were coencapsulated with EBV-immortalized B cells (acting as APCs) stained with CTV. Flow cytometry scatter plots show the gating strategy for T cells (PE+), APCs (CTV+), and NFAT-eGFP reporter activation. The left plot shows the coencapsulation situation of T cells and EBV B cells, with minimal activation detected (Q2: 2.44%) for T cell-only DEs (middle). The right plot shows a higher activation level (Q2: 11.6%) when T cells are coencapsulated with APCs, indicating successful direct immune cell activation within DEs.

Commercial FACS instruments are typically equipped with multiple lasers and detectors, and thus superior to FADS systems for sorting heterogeneous droplet populations. To explore this capability further, we employed three different fluorophores, CTFR (emission at 661 nm), CTV (emission at 450 nm), and CFSE (emission at 517 nm), to label different cell types. Labeled cells were coencapsulated, and the resulting DEs analyzed using a FACS Aria III instrument. Significantly, we were able to discern DEs that contain three different cell types (Figure 4a) and subsequently sort the corresponding DEs (Figure 4b), indicating the potential of our DE platform to study complex cell interactions. More examples of sorted DEs can be found in Figure S11.

Next, we applied the DE platform to an immunological problem of relevance. Specifically, we asked whether single T cells can become specifically activated by antigen-presenting cells in droplets and can then be sorted for further analysis. To test this, we generated Epstein–Barr virus (EBV)-specific CD4^+^ T cell lines from the blood of a healthy donor and transduced T cells with an activation reporter (NFAT-eGFP). As APCs, we used EBV-immortalized B cells from the same donor. Prior to encapsulation, T cells were labeled with anti-CD2-PE antibody and B cells with CTV. Next, cells suspensions were coflown at a concentration of 8 million/mL from two separate syringes to prevent cell-cell interactions (T cell activation) prior to encapsulation. Droplets were collected in Eppendorf tubes and incubated at 37 °C for 16 h. Next, droplets were double emulsified and analyzed by flow cytometry. As shown in Figure 4c, we first gated on DEs containing only T cells. As expected, T cells expressing eGFP are minimal (gated 2.44%, self-stimulation possible), indicating that they were not activated. In contrast, when we gated on DEs containing both an APC (CTV) and a T cell (PE), we found that in 11.6% of DEs T cells expressed eGFP. These data demonstrate that our DE platform is suitable for high-speed (kilohertz rates) identification and isolation of droplets containing T cells directly activated in droplets. In comparison, the only reported droplet TCR T cell screening platform operated at a frequency of ∼0.001 Hz and took approximately fifteen minutes to sort a single droplet.^70^ The ability to sort DEs using multiple channels of a conventional FACS instrument opens up new possibilities for studying complex cellular interactions and identifying rare cell populations at unprecedented efficiency. This, in turn, paves the way for groundbreaking discoveries in cellular biology and immunology.

## Conclusions

We have developed a robust, yet user-friendly workflow tailored for high-throughput screening of cocultured droplets, designed with cell biologists in mind. This platform enables simple confirmatory assays of biological processes, from droplet to result, without requiring extensive microfluidic expertise. Throughout the design and evaluation phases, we addressed a range of potential use scenarios and challenges, including experimental extensibility, DE stability, single DE dispensing, emulsion breakage, and fluorescent labeling efficiency, to ensure our system meets the diverse and evolving needs of laboratory environments. Through the modular design of DE generation, our system not only facilitates easy operation but also allows for the encapsulation of larger contents without compromising DE detection rate. The workflow is flexible, streamlined, and efficient, allowing cell biology laboratories to perform complex multicellular and multisignal droplet sorting experiments with existing flow cytometers using just three additional simple syringe pumps and two easily accessible microfluidic devices. Such experiments were previously only feasible in specialized microfluidic laboratories or by using costly, feature-limited commercial droplet sorters.

Looking ahead, the analysis of biological systems at the single-cell level is essential to understanding function. In recent years, there have been tremendous efforts toward developing systems that allow for the analysis multiple biomarkers from single cells using flow cytometry or CyTOF (cytometry by time-of-flight).^71^ These techniques are now being used in cell biology laboratories and have led to significant discoveries.^72,73^ Similarly, the development of DB scRNA-seq technology has resulted in a proliferation of single-cell transcriptome studies, highlighting the effectiveness and widespread utility of this technology set. However, to date, there is no single technique that can efficiently analyze single-cell interactions. Such a technique, especially if easy to implement, should find numerous applications in cell biology. In our proof-of-principle experiments, we have demonstrated that different single cells can be cocultured in droplets and subjected to multisignal screening, allowing for the analysis of interactions between T cells and APCs. Such a workflow enables the rapid identification of TCRs and their respective peptide antigens, speeding up the development of immunotherapies. Additionally, single-cell coculture workflows can be applied to study almost all interactions between mammalian cells or microbial interactions in coculture. With the broad applicability and simplicity of the methods we describe here, the powerful tool of microfluidic droplets has the potential to open a new chapter as a routine toolbox for the cellular laboratory.

## Data Availability

Raw data including CAD file for chip designs, FCS files for DE sorting, and microscope images and high-speed camera images are available free of charge at ETH Research Collection repository (10.3929/ethz-b-000658790).

## Supporting Information Available

Project background; Extended cell preparation methods; Extended device fabrication and operation; Extended methods for DE sorting and analysis; Chip design; Thin-shell DE advantages; DE stability tests; Flow cytometry analysis; Distinguishing the number of internal droplets; Disruption methods of DE drops; Osmotic pressure manipulation; Comparison of surface labeling and whole cell staining; Supplementary multicolor DE sorting (PDF)
Cell coencapsulation into picoliter droplets, Video S1; conversion of droplets into thin-shell DEs for FACS sorting, Video S2; doublet DE drops passing through an alternating electric field on a microfluidic chip, Video S3 (MP4)
