# Supplementary material for: Robust Double Emulsions for Multicolor Fluorescence-Activated Cell Sorting
Source: Anal Chem. 2024 Sep 4;96(37):14809–18. doi: 10.1021/acs.analchem.4c02363 (PMC11411495; doi:10.1021/acs.analchem.4c02363)
Supplement: Supplementary file 1 — ac4c02363_si_001.pdf [file ac4c02363_si_001.pdf]

Supporting Information

Robust double emulsions for multicolor fluorescence-activated cell sorting

Yun Ding,*[,1] Giada Zoppi,[2] Gaia Antonini,[2] Roger Geiger,*[,2,3] and Andrew J. deMello*[,1]

[1]Institute for Chemical and Bioengineering, Department of Chemistry and Applied Biosciences, ETH Zürich, 8093 Zürich, Switzerland.
[2]Institute for Research in Biomedicine, Faculty of Biomedical Sciences, Università della Svizzera italiana, 6500 Bellinzona, Switzerland.
[3]Institute of Oncology Research, Faculty of Biomedical Sciences, Università della Svizzera italiana, 6500 Bellinzona, Switzerland.

*Yun Ding (yun.ding@chem.ethz.ch), Roger Geiger (roger.geiger@irb.usi.ch), and Andrew J. deMello (andrew.demello@chem.ethz.ch).

Table of Contents

1. Project background

2. Extended cell preparation methods

3. Extended device fabrication and operation

4. Extended methods for DE sorting and analysis

5. Chip design

6. Thin-shell DE advantages

7. DE stability tests

8. Flow cytometry analysis

9. Distinguishing the number of internal droplets

10. Disruption methods of DE drops

11. Osmotic pressure manipulation

12. Comparison of surface labeling and whole cell staining

13. Supplementary multicolor DE Sorting

14. References

1. Project background

The development and application of the analytical workflow described herein was a result of a collaboration between the microfluidics researchers at ETH Zurich (YD and AdM) and immunology researchers at the IRB Bellinzona (GZ, GA and RG). The IRB co-authors identified several limitations in existing droplet sorting and double emulsion (DE) sorting workflows, including the requirement for bespoke and expensive instrumentation, operational complexity, workflow inflexibility, and difficulties associated with multicellular sorting. Accordingly, the primary research objective was to establish a user-friendly workflow for DE sorting that addresses the aforementioned limitations.

The ETH researchers iteratively refined the analytical workflow based on continuous feedback from the IRB researchers, to realize a method that is both effective and accessible. The resulting workflow presented in the manuscript is now routinely used by cell biologists at the IRB. Indeed, all application-based data presented in the manuscript were independently conducted at the IRB.

The following resources were acquired by the IRB to establish the fluorescence-activated cell sorting workflow:

- Single layer microfluidic chips for droplet generation and DE conversion (ETH Zurich or any appropriate third-party)
- Three low pressure neMESYS syringe pumps (CETONI, Jena, Germany)
- MotionBlitz EoSens® mini1 high-speed camera (Mikrotron, Unterschleissheim, Germany)
- QX200™ Droplet Generation Oil for EvaGreen (#1864006, Bio-Rad, Hercules, USA)
- Masterflex™ Tygon™ tubing (ND-100-80, ID 0.25 mm, OD 0.76 mm, Fisher Scientific, Reinach, Switzerland)
- Syringe needles (METCAL 927050-TE, Gauge 27 TE tip, Distrelec, Nanikon, Switzerland)

2. Extended cell preparation methods

Cell culture

Cells were cultured at 37°C in a humidified atmosphere containing 5% CO_2. Unless specified otherwise, cell culture reagents were obtained from Gibco, Thermo Fisher Scientific, USA. RPMI-complete medium and DMEM-complete medium were generated by supplementing RPMI or DMEM, respectively, with 2 mM glutamine, 1% (vol/vol) non-essential amino acids, 1% (vol/vol) sodium pyruvate, 1% (vol/vol) kanamycin, penicillin (50 U/ml), streptomycin (50 µg/ml) and 10% heat-inactivated fetal bovine serum (FBS). IMDM-complete medium was generated by supplementing IMDM with 1% (vol/vol) kanamycin, pen-strep (100 U/ml) and 10% FBS.

Cells

Blood from healthy donors was obtained from the Swiss Blood Donation Center of Lugano and used in compliance with the Federal Office of Public Health (Authorization 2018-02166/CE 3428). Peripheral blood mononuclear cells (PBMCs) were isolated by Ficoll-Paque density gradient centrifugation (Sigma-Aldrich, Buchs, Switzerland). $CD4^+$ and $CD8^+$ primary human T cells were enriched from PBMCs with magnetic microbeads (Miltenyi Biotec, Adliswil, Switzerland) and cultured in RPMI-complete medium. Raji cells (ATCC, Manassas, USA) and Jurkat D1.1 cells (ATCC) were cultured in RPMI-complete medium. HEK293T cells (ATCC) were cultured in DMEM-complete medium. K562 cells (ATCC) were cultured in IMDM-complete medium.

Immortalization of primary human B cells with Epstein Barr Virus (EBV)

$CD19^+$ B cells were enriched from PBMCs from healthy donors using magnetic microbeads (Miltenyi Biotec, Adliswil, Switzerland) and cultured in RPMI-complete medium. $3x10^6$ freshly isolated $CD19^+$ B cells were inoculated with EBV (a kind gift from Antonio Lanzavecchia), cultured for 5 days, split and further expanded for 14 days.

Lentivirus production

The pCDH-EF1α-MCS-(PGK-GFP) and pCDH-EF1α-MCS-(PGK-RFP) plasmids (System Biosciences, Palo Alto, USA) were packaged into lentiviral particles, using a second-generation lentiviral packaging system. HEK293T cells were transiently transfected with the packaging plasmid psPAX2 (Addgene #12260), the VSV-G envelope plasmid pMD2.G (Addgene #12259) and pCDH-EF1α-MCS-(PGK-GFP) or pCDH-EF1α-MCS-(PGK-RFP) using polyethylenimine (Sigma-Aldrich, Buchs, Switzerland) diluted in Opti-MEM. Media were changed after 12 hours. After 48 hours supernatants containing viral particles were harvested and concentrated using the Lenti-X concentrator (TakaraBio, Kusatsu, Japan) according to the manufacturer's instructions.

Lentiviral transduction

Raji cells were transduced in 96-well plates (Corning, Corning, USA) (1.5×10^5 cells per well) in RPMI-complete medium. Concentrated lentiviral particles (pCDH-EF1α-MCS-(PGK-RFP)) were added, followed by spinoculation at 800 g for 30 minutes at 32°C. 24 hours after transduction, cells were transferred to 48-well plates (Corning) and cultured in fresh RPMI-complete medium. After two days, RFP^+ cells were analyzed by flow cytometry and sorted using a FACS Aria III cell sorter (BD Biosciences, San Jose, USA).

Primary human T cells were activated with plate-bound anti-CD3 (5 µg/ml, clone TR66 (a kind gift of Antonio Lanzavecchia), anti-CD28 (1 µg/ml, clone CD28.2, BD Biosciences, San Jose, USA) in 96-well Nunc Maxisorb plates (Thermo Fisher Scientific, San Diego, USA) in the presence of IL-2 (400 U/ml, Thermo Fisher Scientific, San Diego, USA). 10^5 T cells were plated per well in RPMI-complete medium. After 24 hours of activation, concentrated lentivirus (pCDH-EF1α-MCS-(PGK-GFP)) was added and T cells were centrifuged at 800 g for 45 minutes at 32°C. 24 hours later, cells were transferred to 48-well plates and cultured in IL-2-containing medium (50 U/ml). Five days post-transduction, cells were analyzed by flow cytometry and sorted using a FACS Aria III cell sorter.

Cell labeling

T cells were stained with anti-human CD2-PE (clone RPA-2.10; Biolegend, San Diego, USA) or anti-human CD45-APC (clone H130; Thermo Fisher Scientific, San Diego, USA) on ice for 30 minutes followed by extensive washing. Raji cells were labeled with either CellTrace Violet (CTV) or Cell Trace Far Red (CTFR), and K562 cells were labeled with CellTrace CFSE. All labels were sourced from Thermo Fisher Scientific, USA. Cells were resuspended at 10^6/ml in PBS 1x and labeled with CTV, CTFR or CFSE (1:3,000 dilution) for 20 minutes at 37°C. Staining was quenched by adding five times the original staining volume of culture medium to the cells. After 5 minutes incubation, cells were centrifuged and resuspended in fresh medium.

Generation of NFAT-eGFP reporter

The sequence encoding for NFAT-eGFP was cloned from the retroviral plasmid pSIRV-NFAT-eGFP (Addgene plasmid #118031) into the lentiviral vector pCDH-EF1α-MCS-(PGK-NeoR) (System Bioscience, CD811A-1) that was modified as follows: to avoid constitutive expression of eGFP, the EF1α promoter was removed using NheI restriction enzyme digestion and the vector re-ligated. To insert the NFAT-eGFP sequence into the pCDH-MCS-(PGK-NeoR), donor and recipient plasmids were digested with BamHI and NotI enzymes. Digested DNA was separated on an agarose gel, excised, and purified using the NucleoSpin Extract II kit (Macherey Nagel, Oensingen, Switzerland). DNA ligation was carried out using T4 DNA Ligase (BioConcept AG, Allschwil, Switzerland) following manufacturer's instructions. All reactions were transformed into DH10B chemocompetent cells (Life Technologies Europe BV, Bleiswijk, Netherlands) and grown on LB+Agar plates containing 100 µg/mL Ampicillin (Roth AG, Arlesheim, Switzerland). Individual colonies were inoculated in liquid culture and plasmid minipreps were performed using NucleoSpin Plasmid kit (Macherey Nagel). Finally, insertion of the correct NFAT-GFP sequence was verified by Sanger sequencing (Microsynth AG, Balgach, Switzerland).

3. Extended device fabrication and operation

Microfluidic device fabrication

Both sets of microfluidic devices (for droplet generation and double emulsion conversion) were fabricated using polydimethylsiloxane (PDMS) following standard soft lithography protocols.[1] The process involved structure design, mask fabrication, controlled exposure of the photoresist (photolithography), PDMS casting, bonding, and surface treatment, enabling the creation of desired channel structures in the PDMS devices. Briefly, a 2D projection of the microfluidic channel pattern was designed using CAD software (AutoCAD 2018, Autodesk, San Francisco, USA). The pattern was then printed onto a thin-film lithography mask by a third-party service (Micro Lithography Services, Chelmsford, UK). SU-8 3025 photoresist (MicroChem, Westborough, USA) was used to create features of different height (33 ± 1 μm for droplet generation chips and 43 ± 1 μm for DE conversion chips) on two silicon wafers by standard photolithography techniques.[2] Briefly, this involved exposing the spin-coated SU-8 molds to UV light through the lithography mask, followed by a development step to remove unexposed regions of photoresist. The SU-8 molds were then exposed to chlorotrimethylsilane (Sigma-Aldrich, Buchs, Switzerland) vapour for two hours to aid the detachment of PDMS replicas in a subsequent step. A 10:1 (wt:wt) mixture of PDMS base and curing agent (Sylgard 184, Dow Corning, Midland, USA) was thoroughly mixed and degassed and then poured over the SU-8 molds to form the top layers (~0.5 cm thick) of the microfluidic devices. After degassing to remove residual air bubbles, the molds and PDMS were cured in an oven at 70°C for two hours to ensure complete crosslinking of the polymer. Afterwards, the cured PDMS layer was peeled off from the molds and diced into individual devices. Inlet and outlet holes were created using a hole puncher (Syneo, Angleton, USA). The layers were then bonded to glass microscope slides (ground edge 45°, Thermo Scientific, Reinach, Switzerland) pre-coated with a 1 mm-thick layer of PDMS. Bonding was achieved by plasma treatment of PDMS surfaces using a plasma cleaner (Zepto Model 1, Diener Electronic, Ebhausen, Germany). This rendered the naturally hydrophobic PDMS temporarily hydrophilic, facilitating contact bonding process. To restore the hydrophobicity of the microchannel surfaces in droplet generation devices, they were placed in an oven at 70°C overnight. Conversely, the surfaces of channels within DE devices should remain hydrophilic. Accordingly, immediately after plasma bonding, a 1% (wt/wt) solution of polyvinyl alcohol (Mw 13,000-23,000, Sigma Aldrich, Buchs, Switzerland) in isopropyl alcohol (Sigma Aldrich) was injected into the microfluidic channels. The device was then left for 5 minutes to allow covalent linkage between the polyvinyl alcohol molecules and the PDMS, and formation of a permanent hydrophilic surface. Any excess solution was aspirated, and the device was placed on a hot plate at 120°C for 15 minutes to ensure a strong union between the PDMS layers.

Microfluidic co-encapsulation

Cells were counted using a FAST-READ 102 hemocytometer (Biosigma, Cona, Italy) and adjusted to the desired concentration in culture medium. Before loading into syringes, cells were filtered through a 40 μm mesh (Falcon cell filter, Fisher Scientific, Reinach, Switzerland) to remove cell aggregates. 1 ml BD Luer-Lok syringes (Fisher Scientific) were used to load the cell suspension (~ 0.3 ml per sample) and 5 ml BD Luer-Lok syringes (Fisher Scientific) were used to load the oil (Droplet Generation Oil for EvaGreen, Bio-Rad, Hercules, USA). To maximize cell viability, we opted not to use cell density gradient media to balance the cell suspension in our workflow.[3] Instead, we arranged the syringes and pump in a vertical configuration for cell sample injection. Additionally, in the absence of a density gradient medium, cells naturally settle within the droplets, increasing the likelihood of cell interactions. It is worth noting that although cells gradually settle in the syringe over time, we observed no evidence of uneven cell encapsulation during a 90-minute period of operation. Masterflex Tygon tubing (ID 0.25 mm OD 0.76 mm, Fisher Scientific, Reinach, Switzerland) was used to connect the syringe needle (Gauge 27 TE tip, Distrelec, Nanikon, Switzerland) and microfluidic device. neMESYS syringe pumps (low pressure module, CETONI, Jena, Germany) were used to inject both oil and cell samples. Co-encapsulation was performed at a rate of 10 μl/min for the oil stream and 3 μl/min for each cell stream. Droplets were collected in another 1 ml BD syringe. During collection, a portion of the oil (in the lower layer) was aspirated off using a needle (Sterican 21G x 4 3/4", B. Braun, Melsungen, Germany) to provide sufficient space for the collection of all droplets. Other biocompatible fluorosurfactants, including RAN Biotech's 008-Fluorosurfactant, Dolomite's PicoSurf and Fluigent's dSurf, were tested for forming both droplets and DEs. All surfactants were used at 2% in Novec 7500 fluorinated oil. Tests (data not shown) indicated that all surfactants were compatible with our double emulsion workflow, with the only difference being small variations in the cell stream flowrate needed to maintain uniform droplet production. This is expected, since surfactants differ in their ability to reduce interfacial surface tension, which will directly impact droplet size.[4]

Microfluidic DE conversion

After generation, droplets were stored for 30 minutes (with the outlet of the syringe facing upwards) before injection into the DE chip. This storage period allowed droplets to rise and accumulate at the syringe tip, ensuring that they entered the DE chip in a closely packed arrangement, thus minimizing the amount of accompanying oil.

The DE buffer, consisting of 2% (wt/wt) Poloxamer 188 (Sigma Aldrich, Buchs, Switzerland) and 1% (wt/wt) Tween 20 (Sigma Aldrich) in 1x PBS buffer (pH 7.4, Fisher Scientific, Reinach, Switzerland), was loaded into a 5 ml syringe. For DE conversion, we utilized the same pump and connection setup as for droplet generation. However, the pump was reoriented in the opposite (vertical) direction to match the adjusted position of the syringe. To prevent droplets from drying out, the tubing used for droplet injection was pre-coated with oil. This coating helps to prevent the transfer of oil away from the droplets, ensuring their stability and minimizing the likelihood of droplet drying and breakage. The droplet flowrate was varied between 1 and 4 µl/min and the droplet-to-buffer flowrate ratio was maintained at ratio of 1:4. An excessively slow buffer flowrate resulted in multiple droplets in a DE, while excessively high flowrates increased the likelihood of droplet splitting at the repacking orifice.

4. Extended methods for DE sorting and analysis

DE sorting into well plate

DEs in DE buffer were diluted 1:5 with PBS in a 12 × 75 mm round bottom FACS tube (BD Biosciences, San Jose, USA). DEs were gently resuspended by manual swirling of the tube. To maximize DE sorting efficiency, the droplet delay was adjusted from -1.5 to +1.5 delay units (droplet cycles) in increments of 0.25–0.5 delay units. For each delay unit, 1–2 droplets were sorted into different wells of a 96-well plate containing DE buffer. Subsequently, DE drops were imaged using an ImageXpress Micro 4 high-content microscope (Molecular Devices, San Jose, USA) with a 10x objective. One image in both brightfield and fluorescence channels was taken for each well using an exposure time of 200 ms. Finally, wells containing one or more DE drops were manually counted using a benchtop microscope to assess sorting efficiency. All FACS data were analyzed using FlowJo v10 software (BD Biosciences, Ashland, USA) for quantification and assessment of the sorting process.

Droplet imaging

To evaluate co-encapsulation efficiency, primary human T cells expressing GFP and CTV-labeled Raji cells were resuspended in RPMI-complete medium at different concentrations (1×10^6/ml, 2×10^6/ml, 4×10^6/ml, 8×10^6/ml and 15×10^6/ml) and co-encapsulated (oil flowrate = 10 µl/min, cell flowrate = 3 µl/min). Encapsulation was monitored on an E800 upright microscope (Nikon Instruments, Shinagawa, Japan) with a 4x objective, bright-field illumination and MotionBlitz EoSens mini1 high-speed camera (Mikrotron, Unterschleissheim, Germany). To capture higher levels of detail, additional tests were conducted using a Nikon Eclipse Ti-E Inverted microscope (Nikon Instruments) equipped with a Phantom Miro M310 high-speed camera (Vision Research, Wayne, USA). Following co-encapsulation, imaging of W/O droplets was performed using a TE300 inverted microscope (Nikon Instruments, Shinagawa, Japan) with a 10x objective, bright-field illumination and acquired using a Hamamatsu C9100 EM-CCD camera (Hamamatsu Photonics, Hamamatsu City, Japan). Four representative brightfield images were taken for each condition and analyzed using ImageJ/Fiji (https://fiji.sc). To count the total number of droplets, images were first subjected to a "*Gaussian Blur*" smoothing filter and subsequently analyzed with the "*find maxima*" function. Droplets containing one or more cells were manually counted using the "*multi-point function*" in ImageJ/Fiji.

External osmolarity

To assess the effects of external osmolarity, GFP-expressing primary human T cells and CTV-labeled Raji cells were separately resuspended in RPMI-complete medium at a concentration of 4×10^6/ml and co-encapsulated. Droplets were then double emulsified. Four sets of DE drops were incubated in 1x, 2x, 4x and 8x of PBS for 1 hour at 37°C. Before and after sorting, DE drops were analyzed with the Nikon TE300 microscope with 10x objective, Nikon Hg fluorescence lamp (Nikon Instruments, Shinagawa, Japan) and Hamamatsu C9100 EM-CCD camera. Images were acquired in the bright field and DAPI channel with an exposure time of 200 ms.

5. Chip design

The three chip designs used are provided below. The original CAD file with exact dimensions is provided separately.

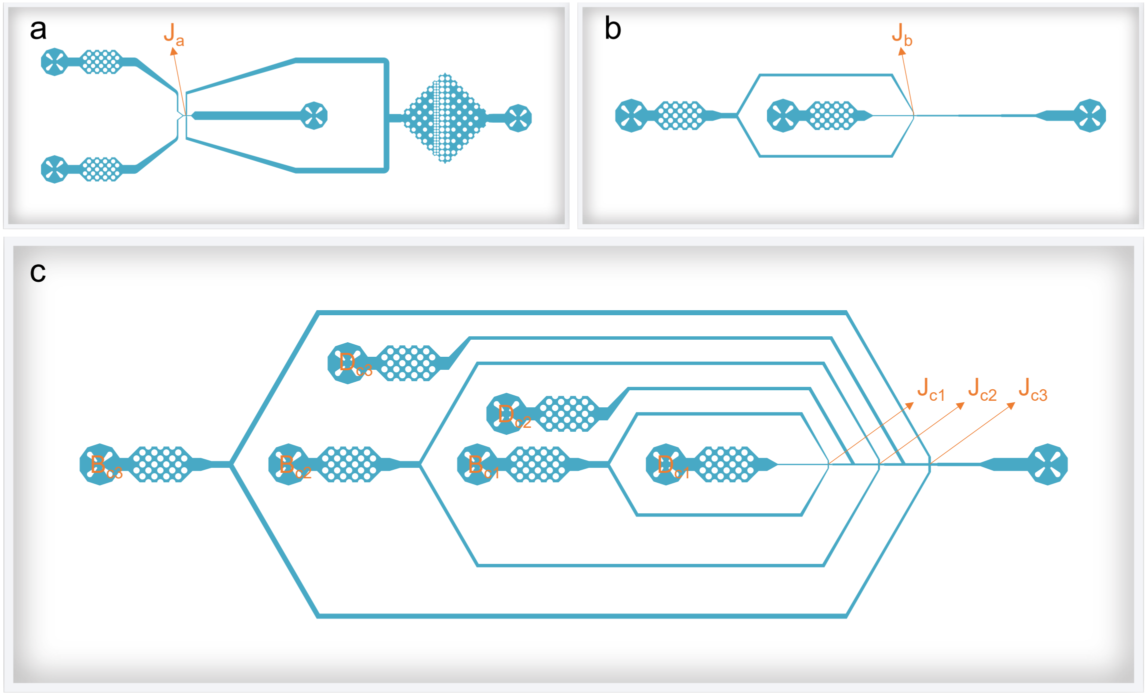

Figure S1. Chip designs for cell co-encapsulation and double emulsion conversion. (a) Cell co-encapsulation chip features a design height of 33 µm with a junction feature size (J_a) of 30 µm. (b) Standard double emulsion conversion chip. Height 43 µm, J_b is 30 µm. (c) Extended double emulsion conversion chip. This chip includes the structure of strandard chip with three sets of droplet and buffer inlets (D_{c1}/B_{C1}, D_{c2}/B_{C2} and D_{c3}/B_{C3}) to accommodate a larger range of input droplet sizes (35–75 µm). Height 43 µm, J_{c1} 30 µm, J_{c2} 45 µm, J_{c2} 60 µm.

6. Thin-shell DE advantages

Thin-shell DEs offer the advantages of facilitating material exchange across the oil layer and providing stronger hydrodynamic resilience.[5–8] These benefits are summarized in **Figure S2**.

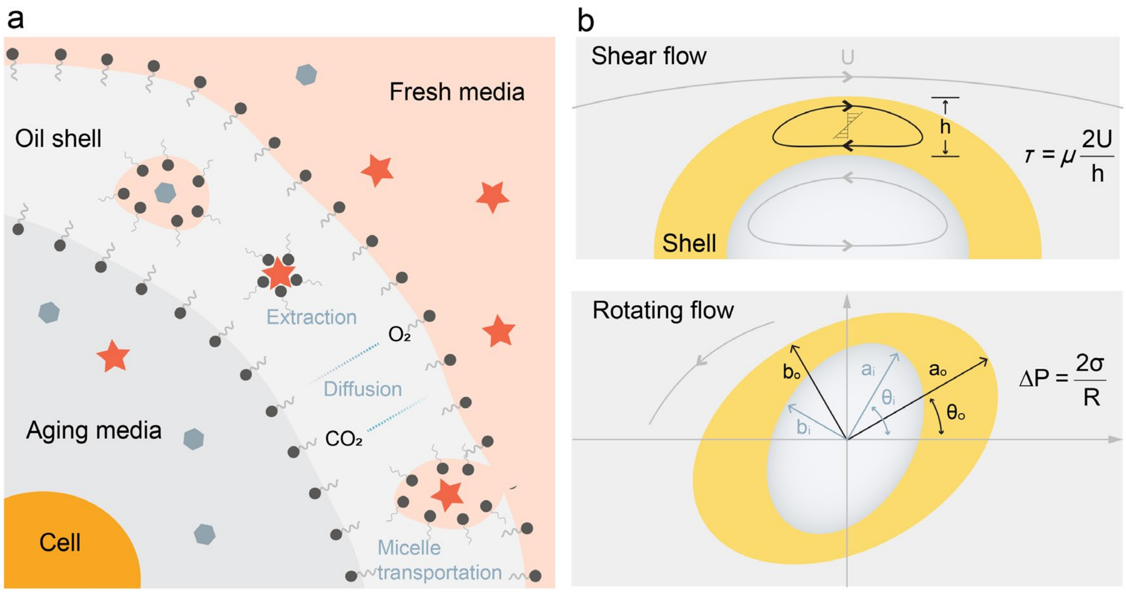

Figure S2. Molecular transport and hydrodynamic properties of DE drops. (a) Mechanisms for molecule transport across the DE oil shell include simple diffusion, surfactant-based chemical extraction, and surfactant-assisted transportation.[5] A thin shell accelerates mass transfer by shortening the transit distance and increasing the concentration gradient. Panel adapted with permission from the Royal Society of Chemistry.[5] (b) Hydrodynamic models for DE drops in shear and rotating flows. Within shear flows, internal circulating flows are formed in the oil shell (yellow zone) and water core (white zone) of a DE drop, and caused by friction between the immiscible phases.[6,7] The shear stress, τ, in the shell is inversely related to the thickness, h, while the flow velocity along the boundary and the dynamic viscosity of the liquid are represented by U and μ, respectively. In rotational flows, a deflection angle is created between the oil shell and water core, resulting in a Laplace pressure (ΔP) "shock".[7,8] The surface tension of the interface and the radius are denoted by σ and R, respectively. Bottom panel adapted with permission from the Royal Society of Chemistry.[8]

7. DE stability tests

Cell-laden DEs showed good stability during storage and exposure to mechanical disturbances. DEs stored for a period of 15 months exhibited negligible rupture. DE resilience to acute mechanical stress was further assessed by vortexing DEs at maximum speed for one minute using a Mixer Vortex Genie 2 in an Eppendorf tube. DEs remained intact after this disturbance test. Detailed results from these stability tests are presented in **Figure S3**.

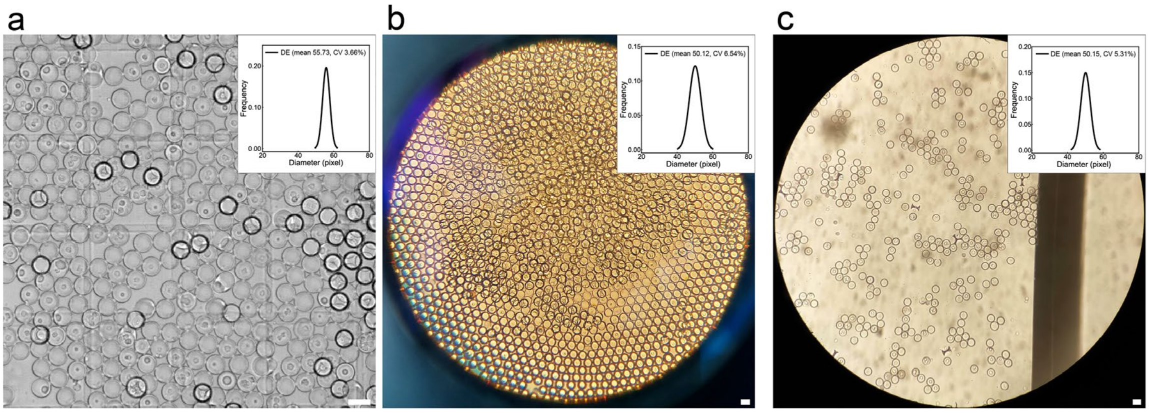

Figure S3. Assessment of the durability and mechanical stability of cell-laden DEs. (a and b) DE storage stability over 15 months. Cells were fixed in 4% formalin prior to encapsulation within DEs, which were stored initially at 4°C for three months and then room temperature for twelve months. DE diameter distributions are shown for (a) freshly formed DEs (CV 3.66%) and (b) aged DEs after 15 months (CV 6.54%). Overlapping DEs and DEs at image edges were excluded from analysis in (b). (c) Mechanical resilience of DEs post-vortexing. DEs containing live cells were subjected to intense vortexing for one minute at a power setting of 10 (maximum speed) on a Mixer Vortex Genie 2. The DE diameter distribution post-vortexing exhibited a CV of 5.31%. Images in (b) and (c) were captured through the eyepiece of a microscope using a smartphone camera. Due to lens distortion, extracted CV values in phone images are likely to be overestimated. Scale bars in all images are 50 µm.

We evaluated DE stability to ionic strength variations. Specifically, we replaced the original DE buffer with fluids of variable osmotic pressure: distilled water (hypotonic), PBS and cell media (isotonic), and 10x PBS (hypertonic), as detailed in **Figure S4**. Data indicated excellent DE stability under both isotonic and hypertonic conditions. Interestingly, whilst internal droplets within DEs shrank when using 10x PBS, this did not compromise DE stability. Conversely, in distilled water, DEs were swelled but remained intact. However, it was noted that when droplets included 16% OptiPrep, a common additive used to control cell density, over half the DEs burst (**Figure S8c**). Given the obvious stability of DEs across various media, we propose substituting external buffers with the relevant cell culture medium for prolonged cell cultivation. Previous studies have identified three primary mechanisms for material exchange across the oil layer in DEs, namely diffusion, surfactant-based chemical extraction, and surfactant-assisted transport.[5] Given its thin oil shell, our DE system is likely to enhance such material exchanges, potentially facilitating more extended cell culture periods. However, further research is required to conclusively determine these benefits.

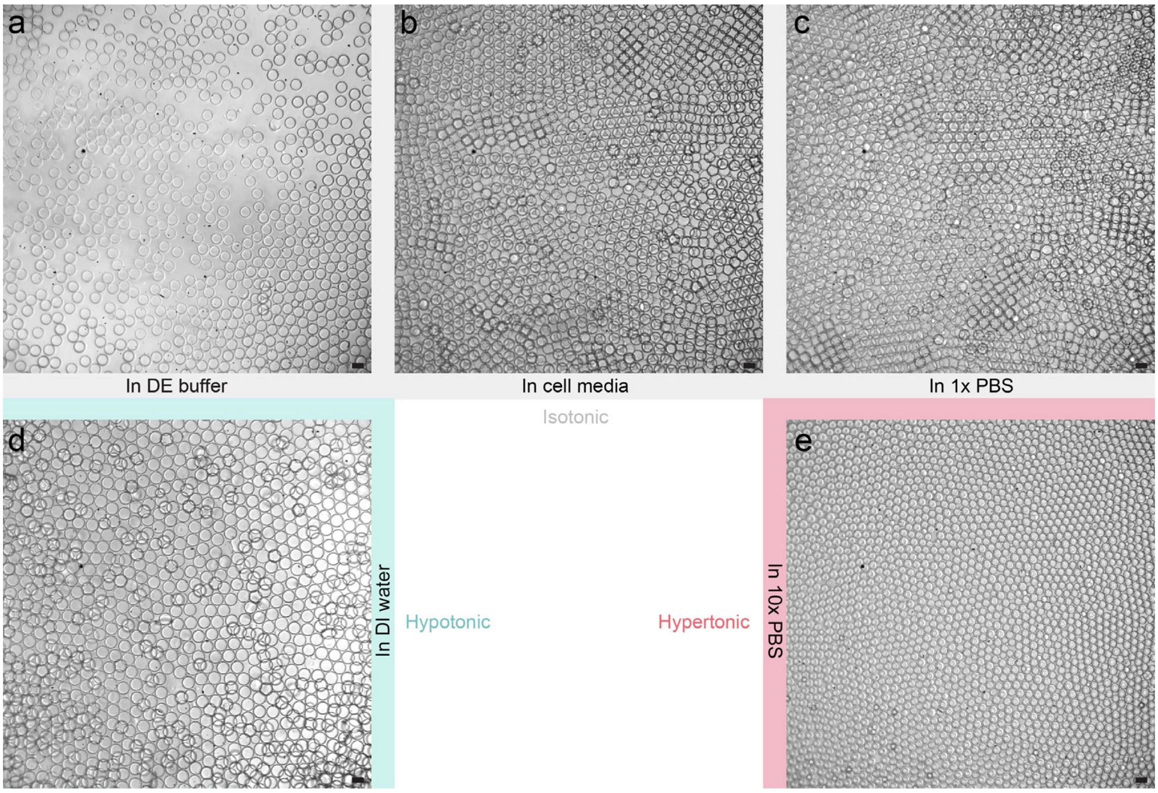

Figure S4. Incubation stability test. DE drops (contained cell media) are incubated in different osmotic concentrations. (a–c) Isotonic: original DE buffer, cell media and 1x PBS. (d) Hypotonic: DI water. (e) Hypertonic: 10x PBS. No breakage is observed in all test environments. The scale bars in all images are 50 μm.

To evaluate the stability of DEs under the application of electric fields, we assessed DE-protected droplet merging events in a microfluidic channel. As shown in Figure S5, paired droplets (one containing PBS the other containing 10% blue ink) in a DE were passed through a microfluidic channel. No droplet merging events were observed as a function of voltage and frequency within the tested range (100-1000 V, 1-10 kHz). For comparison, we also assessed the merging of "normal" droplets under similar electric field conditions in a microfluidic channel (**Figure S5**). More in depth analyses of (microfluidic) droplet merging in electric fields can be found elsewhere.[9,10]

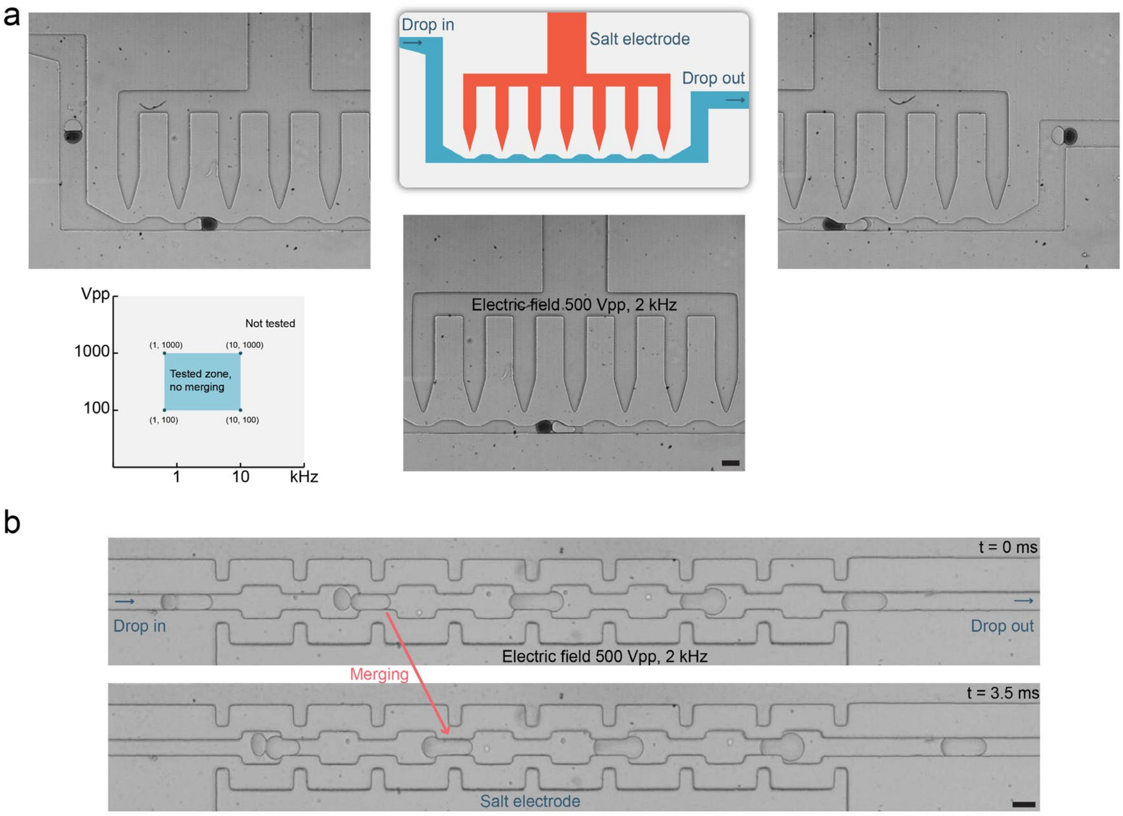

Figure S5. DE stability under alternating electric fields. (a) The DE shields contents from an external electric field. DEs consisting of paired droplets pass through a periodically squeezed channel. The schematic illustrates the experimental setup integrating salt electrodes and the DE flow path. Brightfield images present DEs under an applied electric field of 500 Vpp at 2 kHz, with no droplet merging being observed. The associated video is provided as **Video S3**. The map plot shows the tested voltage and frequency ranges for DE shell-protected droplet merging experiments. (b) Normal droplet merging under a similar electric field condition in a microfluidic channel. The sequence of images depicts the merging of normal droplets under an electric field of 500 Vpp at 2 kHz over 3.5 ms. The scale bars in all images are 50 μm.

8. Flow cytometry analysis

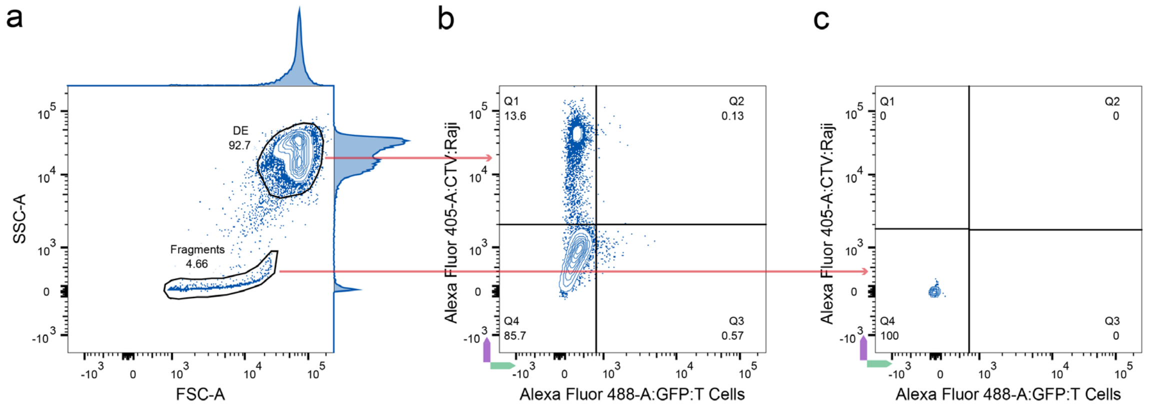

Figure S6. Representative flow cytometry analysis of DEs containing Raji and T cells. GFP-expressing T cells (8 million/ml) and CTV-stained Raji cells (8 million/ml) were co-encapsulated and double emulsified. (a) FSC-SSC scatter plot reveals two distinct populations, with the majority consisting of DEs (92.7%) and a small percentage representing fragments or debris (4.66%). (b) Scatter plot gated on DE events, displaying CTV (Raji cells) vs. GFP (T cells). Note that the GFP signal from T cells in DEs was much weaker compared to CTV staining. (c) Scatter plot gated on "fragment" events, with CTV (Raji cells) vs. GFP (T cells). Fragment populations did not exhibit appreciable fluorescence.

9. Distinguishing the number of internal droplets

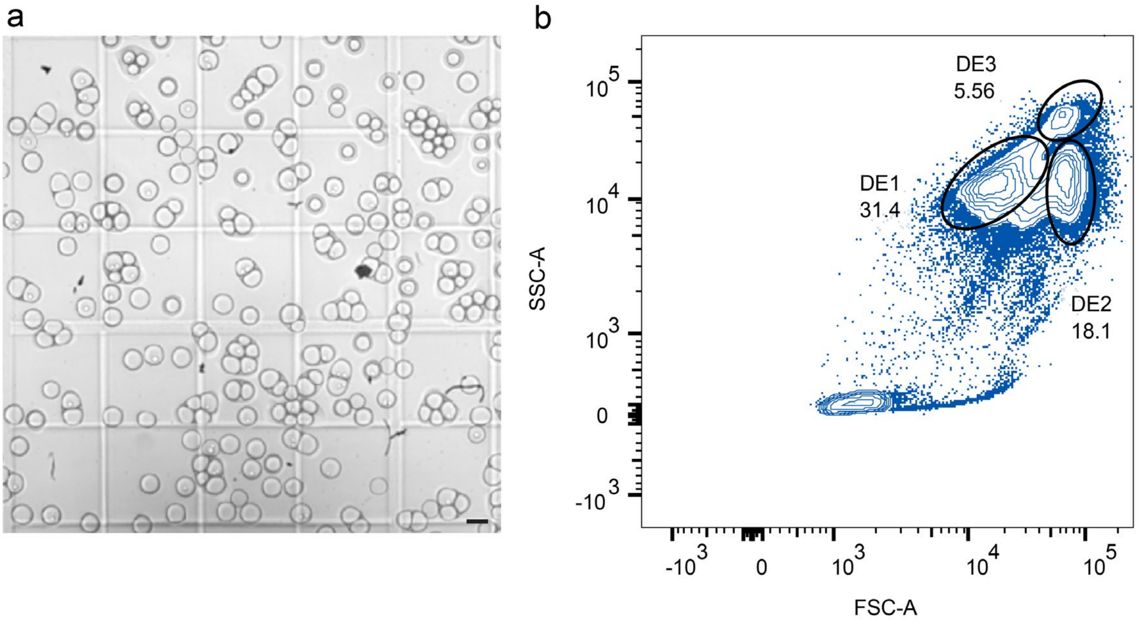

Figure S7. Distinguishing complex double emulsions by examination of their scattering parameters. We produced 'Chaos' DEs by altering the flow rates of the re-encapsulation buffer, resulting in a diverse mixture of DEs with varying numbers of encapsulated cores. (a) Image of these complex DEs. (b) A FACS scatter plot reveals three sub-DE populations. Analysis of the scattering parameters, as well as reference counts, allows for the inference that DE1 is single-core, DE2 is double-core (potentially containing some triplets), and DE3 is multi-core. The scale bar in the image is 50 μm.

10. Disruption methods of DE drops

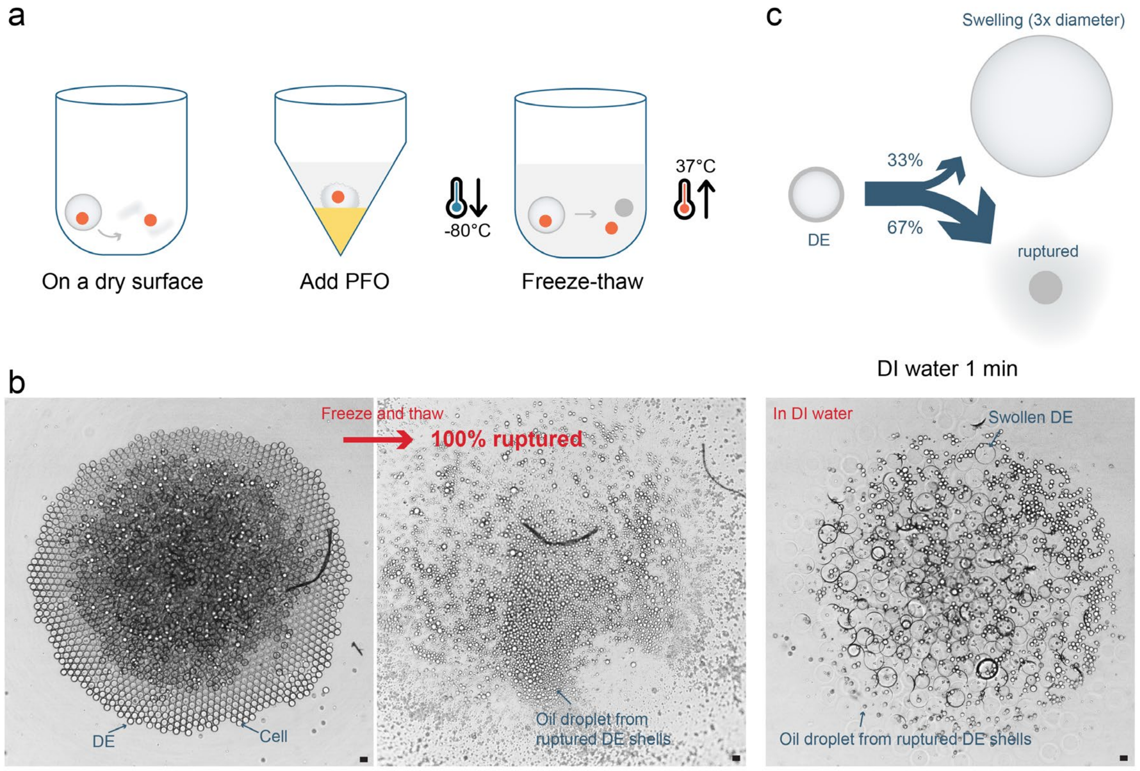

Figure S8. Methods for breaking DE drops. (a) Schematic representation of three effective methods for breaking DE drops in microplate wells, including leaving DEs on a dry surface, adding perfluorooctanol (PFO), and subjecting them to a freeze-thaw process (-80°C to 37°C). (b) The microscopic effect of the freeze-thaw process on the rupture of DE drops. The left image shows intact DE drops with encapsulated cells before temperature treatment. The right image shows that all DE drops are ruptured after freeze-thawing, leaving only oil droplets from ruptured DE shells. (c) Swelling and rupture of DEs in DI water. The DEs are supplemented with 16% OptiPrep. The microscope images show DEs in DI water after 1 minute, with swollen DEs (3x in diameter) and oil droplets from ruptured DE shells visible. Image analysis indicates that approximately 67% of all DEs rupture. Without extra hyperosmotic handling, Drops will not break despite swelling (data shown in **Figure S4d**). The scale bars in all images are 50 µm.

11. Osmotic pressure manipulation

Osmotic pressure control is an effective method for altering DE size post-formation.[11] A disparity in osmotic pressure between the internal and external aqueous phases will initiate water transport across the oil shell of a DE, leading to either DE shrinkage or swelling.[12,13] Water transport mechanisms encompass direct diffusion through the oil layer and transport via "reverse micelles"; the latter being predominant structures formed by surfactants in the oil phase.[14,15] We hypothesized that reducing the internal water core might boost the chances of two-cell interactions. Interestingly, a recent study by Zhuang et al. also suggested that DE shrinkage can be used to increase biomarker concentration in the core, thereby enhancing signal detection.[16] To assess the feasibility of implementing DE contraction and its impact on FACS detection performance, we divided a batch of cell-laden DEs into four groups and replaced their buffers with solutions of 1x PBS, 2x PBS, 4x PBS, and 8x PBS, respectively.

As expected, significant shrinkage occurred. Compared to 1x PBS, DEs in 8x PBS exhibited a 30% reduction in their final diameter (**Figure S9e** and **f**). Although water migration across DEs can occur over several hours,[12] employing a thin oil layer significantly accelerates this process.[17] Consequently, in the current experiments, migration was swift, taking only a few minutes. FACS analysis shows how varying osmotic pressures affect DE populations (**Figure S9a**–**d**). With a decrease in DE size from 1x to 8x PBS, we observed a corresponding drop in the scatter-gated DE population from 92.3% to 69.1%. This reduction is likely due to difficulties in distinguishing contracted DEs from empty oil droplets and other debris. In contrast to the findings of Zhuang et al.,[16] where signal enhancement in fluorescent cellular secretions was observed with DE contraction, our study revealed no clear correlation between fluorescence detection rates and DE shrinkage. Specifically, CTV and GFP detection rates varied minimally across 1x to 8x PBS concentrations. This inconsistency implies that signals emanating from the cell surface or interior do not become enhanced by DE volume reduction. Accordingly, it is essential to consider the signal source when manipulating DE size, as benefits to signal detectability may not be universally applicable.

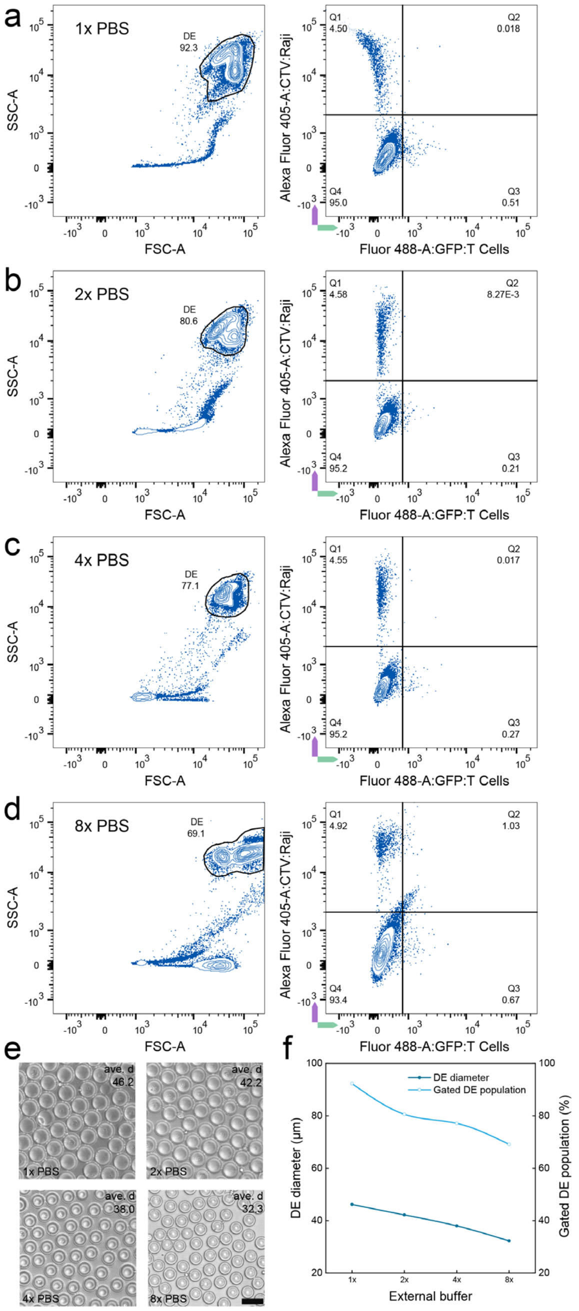

Figure S9. The impact of DE external osmolarity. GFP-expressing T cells (4 million/ml) and CTV-stained Raji cells (4 million/ml) were co-encapsulated and double emulsified before being incubated in PBS buffers of varying concentration. Flow cytometry analysis was then conducted to evaluate the effects of different osmolarities: 1x PBS (a), 2x PBS (b), 4x PBS (c), and 8x PBS (d). The size of the resulting DEs was observed under a microscope (e), and trend plots of DE diameter and gated DE proportion relative to the PBS concentration are shown in (f). The scale bar in the image is 50 μm.

12. Comparison of surface labeling and whole cell staining

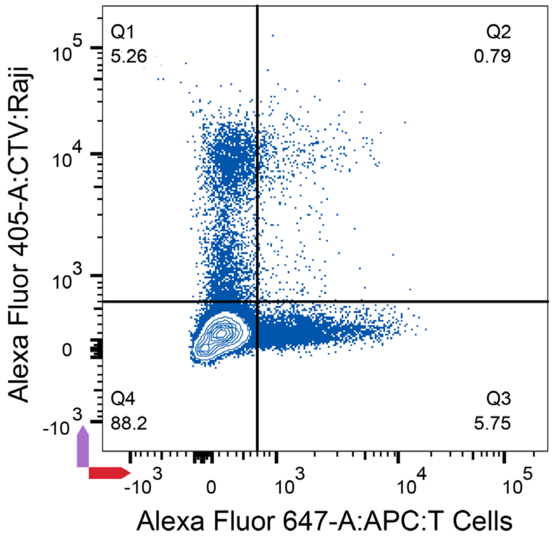

Figure S10. Flow cytometry analysis of co-encapsulated APC-labeled T cells (15 million/ml) and CTV-labeled Raji cells (15 million/ml). The fluorescence gating efficiencies for surface labeling (anti-CD45-APC) and whole cell staining (dye CTV) are found to be similar.

13. Supplementary multicolor DE Sorting

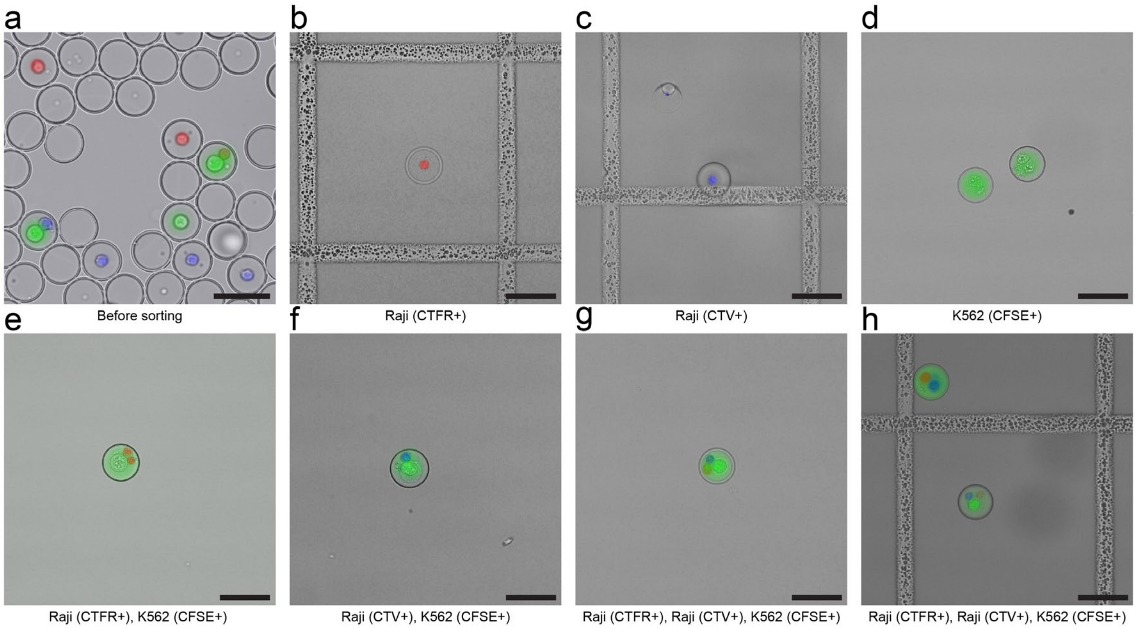

Figure S11. Images presenting multicolor DE sorting. (a) DEs containing a mixture of cell types before sorting. Overlay colors indicate DEs encapsulating Raji cells labeled with CTFR (red) or CTV (blue) and K562 cells labeled with CFSE (green). DEs containing all three cell types were not found in the field of view due to their rarity (less than 0.4% of total events). (b-d) Sorted DEs containing single cell types: Raji cells labeled with CTFR (b), CTV (c), and K562 cells labeled with CFSE (d). (e-h) Sorted DEs demonstrating co-encapsulation of two (e and f) or three (g and h) cell types. The presence of triple-positive DEs post-sorting (h) highlights the power of the method to efficiently enrich rare cell combinations that are challenging to find in the pre-sorted population. The scale bars in all images are 50 μm.

14. References

(1) Cho, S.; Kang, D.-K.; Sim, S.; Geier, F.; Kim, J.-Y.; Niu, X.; Edel, J. B.; Chang, S.-I.; Wootton, R. C. R.; Elvira, K. S.; deMello, A. J. Droplet-Based Microfluidic Platform for High-Throughput, Multi-Parameter Screening of Photosensitizer Activity. *Anal. Chem.* **2013**, *85* (18), 8866–8872. DOI: 10.1021/ac4022067.

(2) Srisa-Art, M.; Kang, D.-K.; Hong, J.; Park, H.; Leatherbarrow, R. J.; Edel, J. B.; Chang, S.-I.; deMello, A. J. Analysis of Protein-Protein Interactions by Using Droplet-Based Microfluidics. *Chembiochem* **2009**, *10* (10), 1605–1611. DOI: 10.1002/cbic.200800841.

(3) Liu, H.; Li, M.; Wang, Y.; Piper, J.; Jiang, L. Improving Single-Cell Encapsulation Efficiency and Reliability through Neutral Buoyancy of Suspension. *Micromachines (Basel)* **2020**, *11* (1), 94. DOI: 10.3390/mi11010094.

(4) Ibrahim, A. M.; Padovani, J. I.; Howe, R. T.; Anis, Y. H. Modeling of Droplet Generation in a Microfluidic Flow-Focusing Junction for Droplet Size Control. *Micromachines (Basel)* **2021**, *12* (6), 590. DOI: 10.3390/mi12060590.

(5) Etienne, G.; Vian, A.; Biočanin, M.; Deplancke, B.; Amstad, E. Cross-Talk between Emulsion Drops: How Are Hydrophilic Reagents Transported across Oil Phases? *Lab Chip* **2018**, *18* (24), 3903–3912. DOI: 10.1039/c8lc01000e.

(6) Leong, C. M.; Gai, Y.; Tang, S. K. Y. Internal Flow in Droplets within a Concentrated Emulsion Flowing in a Microchannel. *Phys. Fluids* **2016**, *28* (11), 112001. DOI: 10.1063/1.4968526.

(7) Tiribocchi, A.; Montessori, A.; Lauricella, M.; Bonaccorso, F.; Succi, S.; Aime, S.; Milani, M.; Weitz, D. A. The Vortex-Driven Dynamics of Droplets within Droplets. *Nat. Commun.* **2021**, *12* (1), 82. DOI: 10.1038/s41467-020-20364-0.

(8) Li, J.; Su, L.; Li, J.; Liu, M.-F.; Chen, S.-F.; Li, B.; Zhang, Z.-W.; Liu, Y.-Y. Influence of Sucrose on the Stability of W1/O/W2 Double Emulsion Droplets. *RSC Adv.* **2015**, *5* (101), 83089–83095. DOI: 10.1039/C5RA11155B.

(9) Ahn, K.; Agresti, J.; Chong, H.; Marquez, M.; Weitz, D. A. Electrocoalescence of Drops Synchronized by Size-Dependent Flow in Microfluidic Channels. *Appl. Phys. Lett* **2006**, *88* (26), 264105. DOI: 10.1063/1.2218058.

(10) Nan, L.; Mao, T.; Shum, H. C. Self-Synchronization of Reinjected Droplets for High-Efficiency Droplet Pairing and Merging. *Microsyst. Nanoeng.* **2023**, *9*, 24. DOI: 10.1038/s41378-023-00502-6.

(11) Mezzenga, R.; Folmer, B. M.; Hughes, E. Design of Double Emulsions by Osmotic Pressure Tailoring. *Langmuir* **2004**, *20* (9), 3574–3582. DOI: 10.1021/la036396k.

(12) Delample, M.; Da Silva, F.; Leal-Calderon, F. Osmotically Driven Gelation in Double Emulsions. *Food Hydrocoll.* **2014**, *38*, 11–19. DOI: 10.1016/j.foodhyd.2013.11.009.

(13) Nollet, M.; Mercé, M.; Laurichesse, E.; Pezon, A.; Soubabère, O.; Besse, S.; Schmitt, V. Water Fluxes and Encapsulation Efficiency in Double Emulsions: Impact of Emulsification and Osmotic Pressure Unbalance. *Soft Matter* **2016**, *12* (14), 3412–3424. DOI: 10.1039/c5sm03089g.

(14) Bahtz, J.; Gunes, D. Z.; Hughes, E.; Pokorny, L.; Riesch, F.; Syrbe, A.; Fischer, P.; Windhab, E. J. Decoupling of Mass Transport Mechanisms in the Stagewise Swelling of Multiple Emulsions. *Langmuir* **2015**, *31* (19), 5265–5273. DOI: 10.1021/acs.langmuir.5b01138.

(15) Garti, N.; Aserin, A. Double Emulsions Stabilized by Macromolecular Surfactants. *Adv. Colloid Interface Sci.* **1996**, *65*, 37–69. DOI: 10.1016/0001-8686(95)00289-8.

(16) Zhuang, S.; Liu, H.; Inglis, D. W.; Li, M. Tuneable Cell-Laden Double-Emulsion Droplets for Enhanced Signal Detection. *Anal. Chem.* **2023**, *95* (3), 2039–2046. DOI: 10.1021/acs.analchem.2c04697.

(17) Bahtz, J.; Gunes, D. Z.; Syrbe, A.; Mosca, N.; Fischer, P.; Windhab, E. J. Quantification of Spontaneous W/O Emulsification and Its Impact on the Swelling Kinetics of Multiple W/O/W Emulsions. *Langmuir* **2016**, *32* (23), 5787–5795. DOI: 10.1021/acs.langmuir.6b00425.
